# Bone Mineral Density in HIV-Negative Men Participating in a Tenofovir Pre-Exposure Prophylaxis Randomized Clinical Trial in San Francisco

**DOI:** 10.1371/journal.pone.0023688

**Published:** 2011-08-29

**Authors:** Albert Y. Liu, Eric Vittinghoff, Deborah E. Sellmeyer, Risha Irvin, Kathleen Mulligan, Kenneth Mayer, Melanie Thompson, Robert Grant, Sonal Pathak, Brandon O'Hara, Roman Gvetadze, Kata Chillag, Lisa Grohskopf, Susan P. Buchbinder

**Affiliations:** 1 San Francisco Department of Public Health, San Francisco, California, United States of America; 2 University of California San Francisco, San Francisco, California, United States of America; 3 Johns Hopkins University School of Medicine, Baltimore, Maryland, United States of America; 4 Fenway Health, Boston, Massachusetts, United States of America; 5 AIDS Research Consortium of Atlanta, Atlanta, Georgia, United States of America; 6 Gladstone Institutes, San Francisco, California, United States of America; 7 Northrop Grumman, Atlanta, Georgia, United States of America; 8 Division of HIV/AIDS Prevention, Centers for Disease Control and Prevention, Atlanta, Georgia, United States of America; Instituto de Pesquisa Clínica Evandro Chagas/Fundação Oswaldo Cruz, Brazil

## Abstract

**Background:**

Pre-exposure prophylaxis (PrEP) trials are evaluating regimens containing tenofovir-disoproxil fumarate (TDF) for HIV prevention. We determined the baseline prevalence of low bone mineral density (BMD) and the effect of TDF on BMD in men who have sex with men (MSM) in a PrEP trial in San Francisco.

**Methods/Findings:**

We evaluated 1) the prevalence of low BMD using Dual Energy X-ray Absorptiometry (DEXA) in a baseline cohort of 210 HIV-uninfected MSM who screened for a randomized clinical trial of daily TDF vs. placebo, and 2) the effects of TDF on BMD in a longitudinal cohort of 184 enrolled men. Half began study drug after a 9-month delay to evaluate changes in risk behavior associated with pill-use. At baseline, 20 participants (10%) had low BMD (Z score≤−2.0 at the L2–L4 spine, total hip, or femoral neck). Low BMD was associated with amphetamine (OR = 5.86, 95% CI 1.70–20.20) and inhalant (OR = 4.57, 95% CI 1.32–15.81) use; men taking multivitamins, calcium, or vitamin D were less likely to have low BMD at baseline (OR = 0.26, 95% CI 0.10–0.71). In the longitudinal analysis, there was a 1.1% net decrease in mean BMD in the TDF vs. the pre-treatment/placebo group at the femoral neck (95% CI 0.4–1.9%), 0.8% net decline at the total hip (95% CI 0.3–1.3%), and 0.7% at the L2–L4 spine (95% CI −0.1–1.5%). At 24 months, 13% vs. 6% of participants experienced >5% BMD loss at the femoral neck in the TDF vs. placebo groups (p = 0.13).

**Conclusions:**

Ten percent of HIV-negative MSM had low BMD at baseline. TDF use resulted in a small but statistically significant decline in BMD at the total hip and femoral neck. Larger studies with longer follow-up are needed to determine the trajectory of BMD changes and any association with clinical fractures.

**Trial Registration:**

ClinicalTrials.gov: NCT00131677

## Introduction

Low bone mineral density (BMD) is common among HIV-infected individuals [1], [2]. The etiology of low BMD in this population is likely multi-factorial, including the effects of chronic HIV infection, antiretroviral therapy, and traditional osteoporosis risk factors (e.g. hypogonadism, low body weight, smoking, and alcohol use) which are prevalent in HIV-infected individuals [3], [4], [5], [6], [7]. Few data exist on BMD in HIV-uninfected men who are at risk for HIV infection. Data on the prevalence and correlates of low BMD in these groups could identify the extent to which low BMD exists prior to HIV infection and better elucidate factors contributing to low BMD in the presence or absence of HIV infection.

There is great interest in using anti-retroviral medication for HIV prevention as pre-exposure prophylaxis (PrEP) in HIV-negative individuals at risk for HIV infection [8], [9]. In November 2010, results from the Global iPrEx trial were released, demonstrating that daily oral emtricitabine/tenofovir (Truvada®) provided 44% additional protection from HIV infection in men who have sex with men (MSM) who were provided a comprehensive package of prevention services [10]. In January 2011, the Center for Disease Control and Prevention (CDC) issued interim guidance to health-care providers who may begin to provide PrEP to their at-risk MSM patients [11]. While the Fem-PrEP trial in African women sponsored by Family Health International was stopped early due to futility [12], the Partners PrEP Study [13] and CDC TDF2 Botswana trial [14] demonstrated over 60% efficacy of tenofovir-based PrEP regimens in serodiscordant couples and heterosexual men and women respectively. All current PrEP trials are testing oral tenofovir disoproxil fumarate (TDF) alone or in combination with emtricitabine (FTC). While selected for its favorable safety profile [15], [16], long half-life [17], and penetration into the genital compartment [18], [19], TDF use decreased BMD in HIV-infected patients in randomized clinical trials [16], [20], [21]. Cases of fractures and/or osteomalacia during TDF therapy have been reported [22], [23], [24], [25]. Proposed potential mechanisms include proximal renal tubular toxicity leading to hypophosphatemia [2] or reduction in osteoblast gene expression and function [26]. Evaluating effects of TDF on bone density in studies of HIV-positive individuals is confounded by the effects of HIV and other antiretrovirals used in treatment regimens that are also associated with decreases in BMD [16], [21]. Therefore, the evaluation of BMD in the context of PrEP trials in HIV-uninfected populations provides a unique opportunity to more directly evaluate TDF effects on bone. This is particularly important in the context of TDF use for prevention, where the acceptable risk∶benefit ratio may need to be substantially more favorable than would be considered sufficient for therapeutic use.

The Centers for Disease Control and Prevention (CDC) sponsored a PrEP trial evaluating the safety of daily oral tenofovir among 400 HIV-uninfected men who have sex with men in San Francisco, Atlanta and Boston [27]. To address these important questions of skeletal health in HIV-negative men at risk for HIV infection, a DEXA substudy was conducted at the San Francisco site. In this paper, we characterize the prevalence of baseline low bone BMD among HIV-uninfected MSM eligible for enrollment in this study and determine the effects of TDF on bone density among men followed longitudinally.

## Methods

The protocol for this trial and supporting CONSORT checklist are available as supporting information; see Checklist S1 and Protocol S1.

### Participants

**Figure 1:**
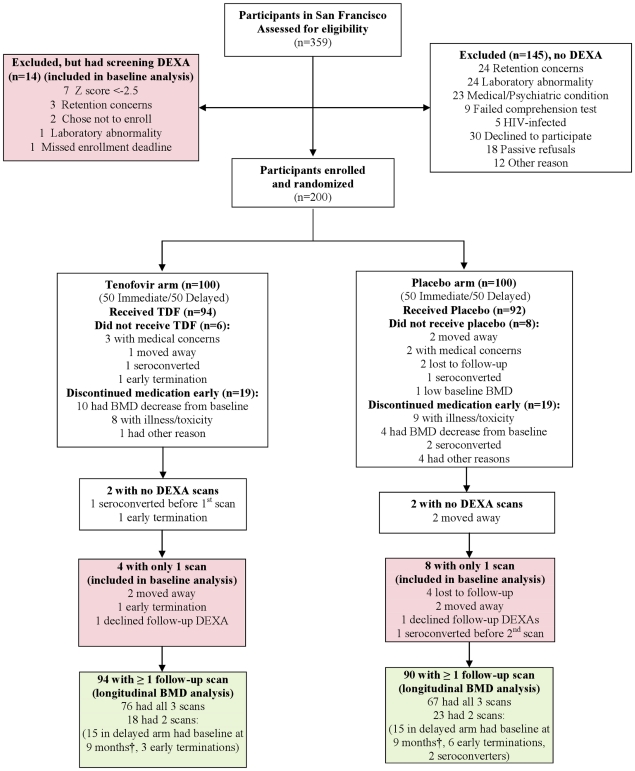
Study design and participant disposition. The ***baseline only cohort*** shown in red shading includes 26 men who had only 1 DEXA scan performed, either during screening or after enrollment. The ***longitudinal*** cohort shown in green shading includes 184 men who had a baseline and at least 1 additional scan during study follow-up. Of the 210 participants who had a baseline DEXA scan, 178 had this scan performed during screening, and 32 shortly after enrollment (prior to the protocol amendment moving DEXA scans to screening). †Delayed arm participants who enrolled prior to protocol amendment had baseline DEXA performed at 9 months prior to starting study drug. DEXA, dual energy X-ray absorptiometry; TDF, tenofovir disoproxil fumarate.

Between February 2005 and July 2007, 200 participants enrolled at the San Francisco Department of Public Health site of the US CDC PrEP study, a phase 2 randomized, double-blind, placebo-controlled extended safety trial of TDF among MSM in the United States (Figure 1). Main inclusion criteria included being male at birth; 18–60 years of age; HIV-1 negative; reporting anal sex with a man in the past 12 months; adequate renal, hepatic, and hematologic function; hepatitis B surface antigen negative; normal urine dipstick or urinalysis; and serum phosphorus, potassium, sodium, and calcium within normal limits. Participants with a history of chronic renal disease; known metabolic bone disease; or current use of nephrotoxic medications or HIV antiretrovirals were excluded.

**Figure 2:**
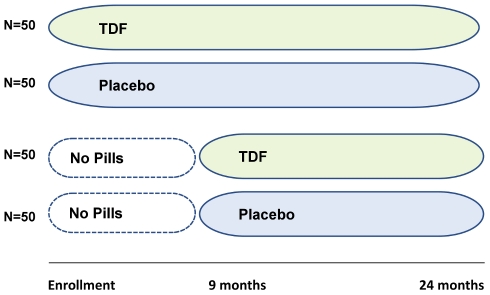
Study design. Participants were randomly assigned to one of four arms. Participants in the 2 immediate arms (TDF vs. placebo) initiated study drug at enrollment; those in the 2 delayed arms (TDF vs. placebo) initiated study drug at the 9 month visit. TDF, tenofovir disoproxil fumarate.

Participants were randomized to one of 4 treatment arms: 1) daily TDF 300 mg beginning at enrollment; 2) daily placebo beginning at enrollment; 3) daily TDF beginning 9 months after enrollment; 4) daily placebo beginning 9 months after enrollment (Figure 2). This immediate vs. deferred treatment design was intended to permit examination of the effects of pill-taking on risk behavior. The study statistician developed the allocation scheme using a permuted blocks randomization scheme in blocks of 8 (2 assignments in each of the 4 arms). Study bottles were filled with TDF or placebo by Gilead Sciences using these codes. Only the study statistician and designated individual at Gilead Sciences had access to treatment assignment codes during the study.

Participants underwent quarterly visits for 2 years, which included assessment of adverse events, rapid HIV testing, laboratory testing for safety monitoring, and a semi-structured questionnaire on sexual and drug use behavior. In the initial version of the protocol, dual-energy X-ray absorptiometry (DEXA) was performed at enrollment, 12 months, and 24 months in the immediate arm to evaluate BMD changes over time. After detection of a number of individuals with low BMD at baseline, a protocol amendment was approved within 9 months of study initiation to conduct baseline DEXA measurements in all eligible individuals during screening, and to exclude individuals with a Z score<−2.5 at the lumbar spine (L2–L4), total hip, or femoral neck and individuals currently receiving treatment for secondary causes of low BMD. DEXA evaluation was not performed in participants found to be ineligible prior to DEXA procedures during the screening process. DEXA scanning was also added for delayed arm participants at 9 and 24 months of follow-up. Study drug was discontinued in participants with a >5% drop from baseline and among HIV seroconverters at the time of first positive rapid HIV test.

Two analytic cohorts are presented in this paper. First, for the analysis of prevalence and correlates of low BMD at baseline, we have included 210 men who had an initial DEXA scan (including 14 men who had a DEXA scan performed during screening but did not enroll in the study). Second, for the longitudinal analysis of TDF effect on BMD, we included 184 men who completed a baseline and at least one DEXA scan during study follow-up.

All participants provided written informed consent prior to study participation. This study was approved by the Institutional Review Boards of the University of California, San Francisco and the CDC.

### Bone mineral density assessment

DEXA scanning of the whole-body, hip, and spine was performed at baseline, 9 months (delayed arm) or 12 months (immediate arm), and 24 months, using a GE Lunar Prodigy densitometer with software version 6.70. The reference population used in the Lunar software are healthy, ambulatory subjects from the general population who had no chronic diseases affecting bone and were not taking medications that affected bone [28]. The database was drawn from studies performed at university medical centers and clinics in the United States, England and Northern Europe. T-scores are based on the reference population ages 20–40; Z-scores were further matched for age, weight, and racial/ethnic group. In accordance with the International Society for Clinical Densitometry 2007 Position Statement on BMD reporting in men younger than age 50 [29], a Z score of ≤−2.0 at either the total hip, femoral neck, or lumbar spine (L2–L4) was considered below the expected range for age.

### Other data collection

Sociodemographic characteristics were collected via interviewer-administered questionnaire at screening. At baseline and quarterly follow-up visits, adverse events (including clinical fractures) and concomitant medications were recorded via clinical interview, and alcohol and drug use were assessed by Audio Computer-Assisted Self Interview (ACASI). Alcohol use was categorized as none, light (1–2 drinks/occasion on no more than 1–2 days/week, or 3–4 drinks/occasion, no more than once a month), moderate (1–2 drinks/occasion on a daily basis or 3–4 drinks/occasion at least 2–3 times/month), or heavy (5–6 drinks/occasion on a daily basis or 6 or more drinks on any one occasion) (adapted from Woody et al [30]). Smoking and exercise patterns and dietary intake of calcium and Vitamin D (Block Calcium/Vitamin D screener [31]) were collected via a one-time interviewer-administered questionnaire conducted during or after study participation. Baseline body weight was calculated using whole-body DEXA data as the sum of total lean, fat, and bone mineral content weights. Laboratory testing, including rapid HIV testing, serum creatinine, phosphorus, and alkaline phosphatase, was performed at each quarterly visit. Creatinine clearance was calculated using the Cockroft-Gault formula [32]. Evaluation of secondary causes was conducted in men with low BMD at screening or during follow-up and those with >5% decrease from baseline at L2–L4 or total hip, including testing for thyroid stimulating hormone, 25-hydroxy vitamin D level, testosterone level, spot urine calcium/creatinine ratio, and serum parathyroid level for participants over age 40.

### Statistical analysis

Mean Z-scores of the lumbar spine (L2–L4), total hip, and femoral neck were calculated, and prevalence of low BMD was determined with exact 95% binomial confidence intervals (CIs). The observed number of low BMD cases was compared using a 2-sided exact binomial test to the number that would have been expected based on reference population data (approximately 2.3% would have Z scores below 2 standard deviations of the mean, assuming Z-scores are normally distributed with mean 0 and standard deviation 1 [33]). The association of sociodemographic variables and risk factors for low bone mass with baseline BMD was examined using univariate logistic regression analysis. Mean percent change in BMD over time was plotted for each anatomic region by treatment arm, and the proportion of men losing >3% and >5% BMD from baseline at 24 months at each site was determined in a pre-specified analysis. These cut-points were chosen because a 3% loss represents more than expected BMD loss in a population of healthy men in which BMD should be stable [34], and a 5% loss corresponds with the approximate BMD loss seen in post-menopausal women over a 2-year period [35].

Linear mixed models with random intercepts were used to assess effects of TDF on percent change in BMD from baseline to 12 and 24 months among immediate arm participants, and from 9 to 24 months among delayed arm participants. Comparison between treatment arms was by intent-to-treat analysis. Preliminary analyses examining potential interactions between treatment assignment (TDF/placebo) and study month, as well as between treatment assignment and arm (immediate/delayed), revealed no interactions. Therefore, immediate and delayed arms were pooled to increase power. All models adjusted for month of follow-up scan (9, 12, or 24) and arm (immediate or delayed). HIV seroconverters were removed from the analysis at the time of first detection of infection. Estimated net treatment differences between the TDF vs. no treatment (either placebo or off-drug period in 1^st^ 9 months of delayed arm) groups with 95% CIs and *P* values for the differences were calculated. A sensitivity analysis was performed censoring participants taken off study drug due to low BMD or >5% decrease in BMD. We also repeated this analysis adjusting for baseline BMD level, age, race/ethnicity, BMI, creatinine clearance, and baseline inhalant (poppers, amyl nitrate, nitrous oxide, or glue) and methamphetamine use. Accounting for the number of visits available for the primary analyses and the observed residual standard deviations and within-subject correlations of the BMD percent loss outcomes, the study had 80% power to detect between-group differences of 0.7 percentage points in L2–L4 and femoral neck BMD loss, and 0.4 percentage points in total hip BMD loss. The linear mixed models were estimated using the xtmixed command in Stata Version 11.2. *P* values<0.05 were considered statistically significant.

## Results

### Study participants

Overall, 359 men were screened for this study in San Francisco, of whom 210 underwent baseline DEXA examination (Figure 1). Of the 200 men who enrolled in the study, 4 did not have any DEXA scans performed; 184 had at least 1 follow-up scan and were included in the longitudinal analysis. The baseline analysis cohort included an additional 26 men who had only one scan performed, comprised of 12 men who enrolled but terminated early or declined further DEXA scans and 14 men who screened but did not enroll. Seven men did not enroll because of low BMD after this criterion was added to the protocol. Baseline participant characteristics of the longitudinal analysis cohort (broken out by TDF vs. placebo) and the additional participants in the baseline-only cohort are shown in Table 1. In comparing the TDF vs. placebo groups, median age, race/ethnicity, smoking, alcohol and recreational drug use, concomitant medication use, dietary calcium and vitamin D intake, exercise patterns, and baseline laboratory parameters did not differ significantly between the 2 groups. Mean weight, total fat mass, and body mass index (BMI), but not fat-free mass, were slightly but significantly higher in the TDF vs. placebo group.

**Table 1:** Baseline characteristics of participants in the baseline only and longitudinal analysis cohorts.

Characteristic	Baseline only(n = 26)†	TDF(n = 94)‡	Placebo(n = 90)‡	2-wayP value*
Age (median, range)	38 (21–59)	40 (19–60)	42 (18–60)	0.91
Race n (%)				
White	18 (69)	76 (81)	67 (74)	
African-American	1 (4)	5 (5)	4 (4)	
Asian/Pacific-Islander	2 (8)	7 (7)	3 (3)	0.10
Latino/Hispanic	2 (8)	5 (5)	9 (10)	
Other (multiethnic, Native American, Middle Eastern)	3 (12)	1 (1)	7 (8)	
Smoking history n (%)				
Nonsmoker		38 (51)	35 (47)	
Former smoker	n/a	18 (24)	27 (36)	0.23
Current smoker (at enrollment)		19 (25)	13 (17)	
Alcohol use in past 3 months†				
No use	2 (17)	15 (16)	13 (14)	
Light use	5 (42)	37 (39)	37 (41)	0.94
Moderate use	4 (33)	38 (40)	35 (39)	
Heavy use	1 (8)	4 (4)	5 (6)	
Medication use (% reporting use during study) Multivitamin, calcium or Vitamin D use				
Corticosteroid use (oral or topical)	11 (42)	60 (64)	53 (59)	0.55
Anabolic hormone use (testosterone, growth hormone)	2 (8)	16 (17)	14 (16)	0.84
Other muscle building supplements (creatine)	0 (0)	2 (2)	2 (2)	1.00
Antidepressants	1 (4)	6 (6)	4 (4)	0.75
	1 (4)	20 (21)	15 (17)	0.46
Recreational drug use (last 3 mo)¥				
Inhalants (poppers, amyl nitrate, nitrous oxide, glue)	4 (33)	26 (28)	34 (38)	0.16
Crack/powder cocaine	2 (17)	12 (13)	11 (12)	1.00
Amphetamines	2 (17)	11 (12)	12 (13)	0.83
Sedatives	0 (0)	10 (11)	14 (16)	0.38
Ecstacy	0 (0)	7 (8)	12 (13)	0.23
Ketamine	0 (0)	1 (1)	2 (2)	0.61
GHB	0 (0)	1 (1)	5 (6)	0.11
Any recreational drug use	4 (33)	41 (44)	47 (52)	0.30
Dietary intake (mean)				
Daily total calcium intake (mg)		725	771	0.43
Daily supplemental calcium intake (mg)	n/a	131	169	0.49
Daily total Vitamin D intake (ug/d)		288	314	0.57
Daily supplemental vitamin D intake (ug/d)		180	196	0.81
Family history of osteoporosis	n/a	11 (14)	6 (8)	0.41
Exercise/Dieting (%)				
Any exercise		64 (84)	62 (83)	0.83
Weight bearing exercise	n/a	64 (85)	62 (83)	0.86
Non-weight bearing exercise		26 (34)	27 (36)	0.87
Dieting in the past 6 mo		20 (27)	19 (25)	1.00
Body composition, mean				
DEXA weight (kg)	**81.9**	**85.8**	**81.5**	**0.05**
BMI (kg/m2)	**25.9**	**27.0**	**25.8**	**0.05**
Total lean mass (kg)	56.4	59.5	59.3	0.85
Total fat mass (kg)	**23**	**23.1**	**19.0**	**0.01**
Laboratory parameters (mean)				
Creatinine (mg/dL)	0.95	0.97	0.97	0.66
Creatinine clearance (mL/min)	123	126	120	0.09
Calcium (mg/dL)	9.5	9.6	9.7	0.69
Phosphorus (mg/dL)	3.6	3.5	3.5	0.94
Serum alkaline phosphatase (g/dL)	75	70	73	0.57
Mean BMD at baseline (g/cm^2^)				
L2–L4 spine	1.12	1.25	1.24	0.86
Total hip	0.99	1.09	1.07	0.48
Femoral neck	0.98	1.06	1.04	0.52

† Baseline only = participants with only 1 DEXA performed; includes 14 screen failures.

‡TDF/placebo = participants randomized to TDF or placebo who had at least 1 follow-up DEXA and were included in the longitudinal cohort.

*P value compares TDF vs. placebo arms of longitudinal cohort.

¥For baseline only group, alcohol and recreational drug use data available for 12/26 participants who enrolled in the study.

**DEXA, dual energy X-ray absorptiometry; TDF, tenofovir disoproxil fumarate; BMD, bone mineral density.**

### Prevalence and correlates of low BMD at baseline

Among the 210 men who received an initial DEXA scan, 20 men (9.5%, 95% CI 5.9–14.3%) had at least one Z-score≤−2.0, with 17 cases at the L2–L4 spine, 5 at the total hip, and 1 at the femoral neck. Prevalence of low BMD by this measure was significantly higher than expected (20 vs. 4.8 cases; p<0.001) under the standard normal assumption. Three individuals had low BMD at 2 anatomic sites, 2 at the total hip and femoral neck, and 1 at the total spine and femoral neck. In univariate analysis (table 2), men who used amphetamines (OR = 5.9, p<0.01) or inhalants (OR = 4.6, p = 0.02) were significantly more likely to have low BMD at baseline. Men who reported supplemental calcium/vitamin D use (59%) were less likely to have low BMD (OR = 0.26, p = 0.009). Because there were only 12 low BMD cases among the 196 men with complete baseline covariate data, multivariable analysis of low BMD was not performed.

**Table 2:** Selected Parameters Associated with Low Bone Mineral Density (Z score≤−2.0).

Characteristic	Univariate OR	*95% CI*	*P*
Age (yrs)	0.98	0.94–1.03	0.46
Race			
White	(ref)		
African-American	1.17	0.14–9.89	0.89
Asian/Pacific-Islander	n/a‡	n/a	n/a
Latino/Hispanic	1.50	0.31–7.28	0.62
Other	3.94	0.94–16.5	0.06
*Tobacco use* *			
Never	(ref)		
Former	1.69	0.40–7.12	0.47
Current	1.11	0.19–6.37	0.91
*Alcohol use (past 3 mo)* †			
None	(ref)		
Light	1.55	0.17–14.4	0.70
Moderate	2.90	0.34–24.6	0.33
Heavy	n/a‡	n/a	n/a
*Medication use*			
Multivitamin, calcium or Vitamin D use	**0.26**	**0.10–0.71**	**<0.01**
Corticosteroid use	n/a‡	n/a	n/a
Anabolic hormone use (testosterone)	n/a‡	n/a	n/a
Other muscle building supplements/compounds	n/a‡	n/a	n/a
Antidepressants	0.83	0.23–3.03	0.79
*Recreational drug use (past 3 mo)* †			
Amphetamine	**5.86**	**1.70–20.20**	**<0.01**
Inhalants	**4.57**	**1.32–15.81**	**0.02**
Cocaine	2.45	0.62–9.76	0.20
Sedatives	0.64	0.08–5.16	0.67
Ecstasy	3.48	0.85–14.16	0.08
GHB	3.24	0.35–30.15	0.30
Daily total calcium intake (per 100 mg increase)	0.97	0.83–1.13	0.69
Daily total vitamin D intake (per 100 IU increase)	0.84	0.62–1.13	0.25
Family history of osteoporosis*	1.02	0.12–8.66	0.99
Any exercise*	0.75	0.15–3.75	0.72
<1 hour/day	1.02	0.12–8.66	0.99
1–2 hours/day	0.53	0.07–4.05	0.55
2–3 hours/day	2.13	0.38–12.03	0.39
>3 hours/day	0.64	0.05–7.62	0.72
Body mass index†	1.11	0.95–1.29	0.17

†BMI, alcohol, and recreational drug use data were available in 196 subjects who enrolled.

‡There were no cases of low BMD among Asian/Pacific Islander men, heavy alcohol users, and those who reported use of corticosteroids, anabolic hormones, or muscle building supplements.

*Tobacco use and exercise/family history data available in 155 participants who enrolled.

### Tenofovir Effect on BMD

At 2 of 3 anatomic locations, TDF exposure resulted in a statistically significant decrease in BMD relative to baseline when compared to the pre-treatment/placebo group. In the intent-to-treat analysis, there was a 1.1% mean net decrease in BMD in the TDF vs. pre-treatment/placebo group at the femoral neck (95% CI 0.4–1.9%, p = 0.004) and an 0.8% net decline at the total hip (95% CI 0.3–1.3%, p = 0.003); at the L2–L4 spine, there was non-significant evidence for an adverse effect (0.7% decline, 95% CI −0.1–1.5%, p = 0.11). After censoring follow-up for individuals taken off study drug due to a >5% drop in BMD or low BMD on a follow-up scan, the net loss was 1.2% (p = 0.002), 0.8% (p = 0.003), and 0.9% (p = 0.039) for the femoral neck, total hip, and L2–L4 spine respectively. Results were similar after adjustment for baseline BMD, BMI, creatinine clearance, race, age, and baseline inhalant and amphetamine use.

**Figure 3:**
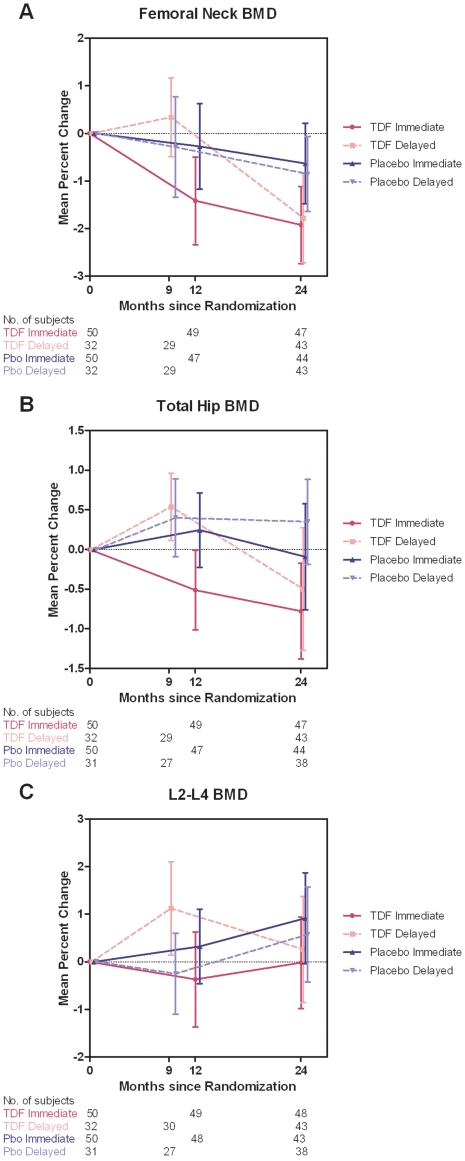
Mean percent change in BMD from baseline at the total spine, total hip, and femoral neck. Trajectory of mean percent change in BMD at the femoral neck (a), total hip (b), and L2–L4 spine (c), by treatment arm. Solid lines represent the immediate arm, and dashed lines represent the delayed arm. Participants who discontinued study drug due to >5% BMD loss from baseline are included. BMD, bone mineral density.

Trajectories of BMD change over time by anatomic site are shown in Figure 3. Declines in BMD in the TDF group were most prominent in the first 12 months of treatment in the immediate arm, with similar decreases seen in the delayed arm upon initiation of TDF during the 9 to 24 month period. Initial BMD declines associated with TDF exposure were most apparent at the femoral neck. Trajectory plots for placebo recipients or TDF delayed-arm participants off study drug during the first 9 month period showed stable or increasing BMD at the total hip and L2–L4 spine and milder (<1%) declines at the femoral neck. In an exploratory analysis evaluating a time by treatment interaction, TDF effects on BMD did not differ at 24 vs. 12 months in the immediate arm. Estimates of the incremental percent bone loss at 24 vs. 12 months were +0.37% (95% CI −0.8–1.5, p = 0.53) at the femoral neck, −0.12% (95% CI −0.9–0.6, p = 0.75) at the total hip, and +0.2% (95% CI −1.0–1.4, p = 0.73) at the L2–L4 spine.

**Figure 4:**
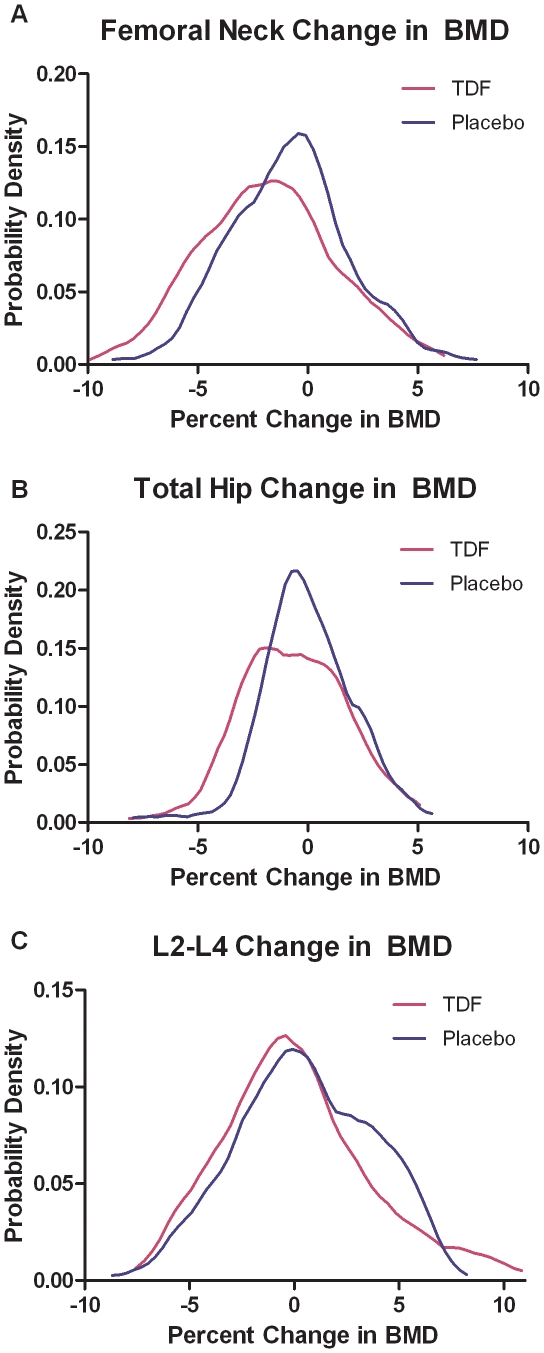
Normal curves demonstrating percent changes in bone density at 24 months in the placebo vs. TDF groups, by anatomic site. Distribution curves of percent BMD change from baseline at last scan at the femoral neck (a), total hip (b), L2–L4 spine (c). BMD, bone mineral density;TDF, tenofovir disoproxil fumarate.

Percent BMD change from baseline in the TDF vs. placebo groups is shown in Figure 4. A greater proportion of participants experienced >3% BMD loss at 24 months at the total hip and femoral neck in the TDF vs. placebo groups. Specifically, 36% vs. 20% lost more than 3% BMD at the femoral neck (p = 0.02), 14% vs. 3% at the total hip (p = 0.02), and 17% vs. 15% at the L2–L4 spine (p = 0.69). Furthermore, 13% vs. 6% participants experienced >5% loss of BMD at the femoral neck in the TDF vs. placebo groups, a difference that was not statistically significant (p = 0.13).

### Evaluation of secondary causes of low BMD

Secondary evaluation of low BMD was performed in 16/20 participants with low BMD at baseline and revealed Vitamin D deficiency in 2 men (25-OH vitamin D level: <4 ng/ml and 11 ng/ml) and hypogonadism (total testosterone = 194 ng/ml) in one individual. Testing was also performed in all 11 cases of >5% loss in BMD from baseline at the total spine or hip and revealed hypogonadism (total testosterone = 230 ng/ml) in one individual.

### Fracture incidence

Among the 184 men enrolled in the longitudinal cohort, there were 10 participants noted to have fractures: 6 participants in the TDF group and 4 participants in the placebo group (p = 0.75). The 6 participants in the TDF group had a total of 8 fractures; the 4 participants in the placebo group had 4 fractures. All fractures were trauma-related and assessed by the investigators to be unrelated to study drug. None of these individuals had a total spine, total hip, or femoral neck BMD Z score≤−2.0 at any time point.

## Discussion

In this study, we found a significant proportion (10%) of healthy HIV-negative MSM at risk for HIV infection had low BMD at baseline. While a number of studies using other classification criteria have shown a higher than expected prevalence of low BMD among HIV-infected MSM, including men with primary HIV infection [36], little data exist on bone mass in healthy MSM without HIV infection. Our findings suggest that some degree of the low BMD observed in HIV-infected men may pre-date HIV infection. We used ISCD classification criteria for BMD reporting in men younger than age 50. These guidelines stipulate that T-scores should not be used and a Z-score of ≤2.0 is defined as “below the expected range for age” [29]; it is emphasized that in men in this age group, osteoporosis cannot be diagnosed on the basis of BMD alone. While the BMD reference ranges for men used in this study may not fully represent the current population studied, men with a Z-score≤−2.0 fall within the lowest 2.5 percentile of BMD compared with the reference population and are typically evaluated for secondary causes of low BMD if their bone density comes to medical attention. In testing 16 such individuals, we uncovered 2 cases of low vitamin D and one case of hypogonadism, suggesting the importance of pursuing a work-up for reversible causes in this population. However, the implications of low BMD for fracture risk in this cohort, particularly among younger men, are currently unknown.

Low BMD was associated with amphetamine and inhalant use in our study, while men reporting use of multivitamins and supplements containing calcium or vitamin D were less likely to have low BMD. A review of the literature shows two reports of a potential association between methamphetamine use and low BMD/altered bone metabolism [37], [38]. The association of methamphetamine and inhalant use with low BMD could be due to a direct toxic effect of these substances on bone metabolism [39], or may be confounded by another lifestyle factor that is associated with both drug use and low BMD. Experimental studies examining potential biological mechanisms for methamphetamine and inhalant-induced bone loss should be conducted, along with larger cross-sectional and cohort studies of drug-using populations to confirm and further investigate these associations. Several studies have found an independent association of amphetamine and/or inhalant use with HIV acquisition among MSM in the United States [40], [41], [42]; this substance-using population may be more likely to benefit from PrEP if effective, but also to demonstrate low BMD at baseline.

Among men who enrolled in this PrEP trial and were included in the longitudinal analysis, TDF use resulted in a small (0.8–1.1%) but statistically significant net decrease in BMD from baseline at two anatomic sites, with the greatest loss at the femoral neck. These changes occurred within the first 12 months of tenofovir use, with no evidence of further declines in BMD at 24 months in the immediate arm, although our sample size limited power to examine differences in the treatment effect across the two time points. We also observed a higher proportion of men experiencing a >5% drop in BMD from baseline in the TDF group relative to placebo, particularly evident at the femoral neck, but this difference was not statistically significant.

Prior studies have demonstrated BMD loss with initiation of ART, regardless of regimen, with somewhat greater decreases observed with tenofovir-containing regimens [6], [16], [21], [43], [44]. Our results are consistent with findings of decreased BMD associated with TDF seen in earlier randomized trials of antiretroviral-naive HIV-infected individuals. The difference in BMD decline associated with TDF compared with placebo in this study is similar in magnitude to the net BMD decline associated with TDF-containing regimens versus alternative regimens in treatment studies. In the Gilead 903 study, Gallant et al. demonstrated a net decrease in BMD of 1.2% at the lumbar spine in the TDF vs. d4T arms; bone loss in the TDF group occurred through weeks 24 and 48 in this cohort and stabilized through week 144 [16]. No additional bone loss was seen in a subgroup of Gilead 903 participants followed through 288 weeks [45]; participants in this open-label extension received supplemental calcium and vitamin D. In the more recent ASSERT trial, Stellbrink et al. reported a net 0.8% and 1.7% decrease in BMD at the lumbar spine and total hip respectively, when comparing the tenofovir-emtricitabine vs. the abacavir-lamivudine group [21]. In this European cohort, bone loss in the TDF group stabilized at week 24 at the lumbar spine but ongoing loss occurred through week 48 at the total hip.

Given the relatively short duration of follow-up in most these studies, longer term BMD data are required to better characterize the long-term effects of TDF on BMD. Current PrEP trials are testing tenofovir-based regimens in over 20,000 HIV-uninfected individuals at risk for HIV infection. Several of these trials are measuring BMD in a subset of study participants, including the iPrEx trial, a phase 3 efficacy trial of emtricitabine-tenofovir in MSM globally. Given our findings, we encourage other PrEP trials to include DEXA monitoring when logistically possible to better characterize the baseline prevalence of low BMD in different target populations for PrEP and the prevalence of risk factors for low BMD, and determine the magnitude and trajectory of BMD loss associated with ARV use for prevention. These data may help identify individuals who are at risk for low BMD or bone loss with PrEP use and guide clinical decision making on whether screening for low BMD may be warranted prior to initiation of PrEP.

The clinical significance of TDF-associated BMD loss, including whether fracture risk is increased, is currently unknown [46]. In this study, we observed 6 fractures in the TDF group vs. 4 in the placebo group, although this study was not designed or powered to detect differences in fracture rates between arms. All fractures were trauma-related and assessed as unrelated to study drug. In the Gilead 903 study, 16 patients (11 in the stavudine group vs. 5 in the tenofovir group) developed fractures through 144 weeks, and almost all were related to trauma [16]. However, there have been case reports of fractures during TDF therapy, in the setting of proximal renal tubule dysfunction. Additional follow-up in larger cohorts is needed to determine whether extended use of TDF increases fracture risk.

Our study is subject to some limitations. First, this study was conducted in only 1 site (San Francisco) in HIV-uninfected men, the majority of whom were white. Additional studies are being conducted in different settings and in other populations, including HIV-uninfected women, and will determine whether our findings can be generalized. Second, we had a relatively small sample size, precluding multivariable analysis of factors associated with low BMD at baseline, as well as analyses to identify any subgroups at higher risk for BMD loss during TDF PrEP use. Also, our prevalence estimate for baseline low BMD was based on a convenience sample of men screening for an HIV prevention study. Therefore, these results may not reflect the prevalence of low BMD in the larger population of MSM. Our study employed relatively short follow-up (maximum 24 months for immediate arm participants). Additional studies are needed to determine whether BMD effects of TDF are sustained or progress during longer term use, and whether these effects reverse after TDF discontinuation. Also, we did not have the opportunity to do more extensive testing for secondary causes of low BMD or to evaluate markers of bone mineral turnover to help elucidate mechanisms for TDF-associated bone loss. In the ASSERT study, increases in bone turnover markers (including osteocalcin, procollagen 1 N-terminal propeptide, bone specific alkaline phosphatase, and type 1 collagen cross-linked C telopeptide) were significantly greater in the TDF vs. comparator group [21]. Future studies should incorporate testing of markers of bone resorption and formation to evaluate potential mechanisms for BMD loss associated with TDF. Finally, these analyses do not adjust for degree of exposure to study drug. Lesser drug exposure due to suboptimal pill-use may have attenuated the magnitude of effects of TDF on BMD we detected in this study. Optimally, such analyses would be adjusted using a biologic marker of long-term drug exposure. Such measures, including tenofovir concentrations in peripheral blood mononuclear cells and hair [47], are currently being explored and should be correlated with bone turnover markers and BMD outcomes in future TDF-based PrEP studies.

Our study also has several strengths. We present novel data looking at prevalence of low BMD in HIV-uninfected men at risk for HIV infection and the effects of TDF on BMD in this seronegative population. These data provide important information on skeletal health in men in the absence of HIV infection and other antiretroviral use. For the longitudinal analysis, this study utilized an intent-to-treat analysis of this randomized, placebo-controlled cohort, thus avoiding confounding by indicator in our assessments. We also achieved high levels of follow-up during the trial.

In summary, we found a significant proportion of HIV-uninfected men had low BMD at baseline. Low BMD was associated with methamphetamine and inhalant use. Similar adverse effects of TDF on BMD were seen in this cohort of HIV-uninfected MSM as seen in antiretroviral treatment studies of TDF-based regimens in HIV-infected individuals. These data suggest that low BMD may pre-date HIV infection among men at risk for acquisition of HIV, and use of tenofovir in these individuals leads to a small but statistically significant decline in BMD. The decline was not associated with an elevated fracture risk during the study.

The finding that oral FTC/TDF PrEP reduces HIV acquisition among MSM [10] and the issuance of interim guidance on prescribing PrEP from the CDC to health-care providers [11] will likely lead to increased PrEP use in different MSM communities. Larger controlled studies with longer follow-up are needed to assess the course of BMD loss associated with tenofovir-based PrEP regimens over the longer term, as well as the clinical significance of these findings in HIV-uninfected populations.

## Supporting Information

Checklist S1 **CONSORT Checklist.**
(DOC)

Protocol S1 **Trial Protocol**
(DOC)
