# Supplementary material for: Bone Mineral Density in HIV-Negative Men Participating in a Tenofovir Pre-Exposure Prophylaxis Randomized Clinical Trial in San Francisco
Source: PLoS One. 2011 Aug 29;6(8):e23688. doi: 10.1371/journal.pone.0023688 (PMC3163584; doi:10.1371/journal.pone.0023688)
Supplement: Protocol S1 — Trial Protocol (DOC) [file pone.0023688.s002.doc]

CLINICAL STUDY PROTOCOL

| PROTOCOL TITLE: | Phase II Extended Safety Study of Tenofovir Disoproxil Fumarate (TDF) among HIV-1 Negative Men |
| --- | --- |
| CDC PROTOCOL NUMBER: | 4323 |
| STUDY DRUG: | Tenofovir Disoproxil Fumarate (TDF) |
| CLINICAL TRIAL SPONSOR: | Epidemiology Branch, Division of HIV Prevention, National Center for HIV, TB, and STD Prevention, Centers for Disease Control and Prevention (CDC), Atlanta, GA |
| PHARMACEUTICAL SPONSOR: | Gilead Science, Inc.  333 Lakeside Drive  Foster City, CA 94404 |
| CLINICAL TRIAL MONITOR: | Constella Group, Inc. |
| CDC PROJECT OFFICERS: | Lisa Grohskopf, MD  Kata Chillag, PhD |
| SITE INVESTIGATORS: | Susan Buchbinder, MD, (SFDPH)  Albert Liu, MD, (SFDPH)  Melanie Thompson, MD (ARCA)  Kenneth Mayer, MD (FCH) |
| PROTOCOL VERSION/DATE | Version 1.6, February 16, 2007 |

TABLE OF CONTENTS

[1 INTRODUCTION 9](#__RefHeading___Toc75597421)

[1.1 Background 9](#__RefHeading___Toc75597422)

[1.2 TDF 10](#__RefHeading___Toc75597423)

[1.2.1 General Information 10](#__RefHeading___Toc75597424)

[1.2.2 Preclinical Pharmacology and Toxicology 11](#__RefHeading___Toc75597425)

[1.2.3 Clinical Trials 12](#__RefHeading___Toc75597426)

[1.2.4 Pharmacokinetics 13](#__RefHeading___Toc75597427)

[1.2.5 Safety 13](#__RefHeading___Toc75597428)

[1.2.6 Efficacy 17](#__RefHeading___Toc75597429)

[1.2.7 Clinical Experience 18](#__RefHeading___Toc75597430)

[1.2.8 Non-Human Primate Challenge Studies 19](#__RefHeading___Toc75597431)

[1.3 Behavioral Disinhibition 20](#__RefHeading___Toc75597432)

[1.4 Rationale for the Current Study 21](#__RefHeading___Toc75597433)

[2 STUDY OBJECTIVES 23](#__RefHeading___Toc75597434)

[3 STUDY DESIGN 23](#__RefHeading___Toc75597435)

[4 PARTICIPANTS 26](#__RefHeading___Toc75597436)

[4.1 Target Populations 26](#__RefHeading___Toc75597437)

[4.2 Number of Participants and Participant Selection 26](#__RefHeading___Toc75597438)

[4.3 Inclusion Criteria 27](#__RefHeading___Toc75597439)

[4.4 Exclusion Criteria 28](#__RefHeading___Toc75597440)

[5 TREATMENT PLAN AND STUDY PRODUCT 31](#__RefHeading___Toc75597441)

[5.1 Treatment Plan 31](#__RefHeading___Toc75597442)

[5.2 Randomization 31](#__RefHeading___Toc75597443)

[5.2.1 Blinding 32](#__RefHeading___Toc75597444)

[5.2.2 Minimizing unblinding related to adverse events 32](#__RefHeading___Toc75597445)

[5.2.3 Minimizing potential for self-unblinding 33](#__RefHeading___Toc75597446)

[5.3 Study Product 34](#__RefHeading___Toc75597447)

[5.3.1 Study Product Handling and Packaging 34](#__RefHeading___Toc75597448)

[5.4 Excluded Concomitant Medications 35](#__RefHeading___Toc75597449)

[6 MAIN STUDY PROCEDURES (PART A) 36](#__RefHeading___Toc75597450)

[6.1 Recruitment 37](#__RefHeading___Toc75597451)

[6.2 Screening Evaluation 37](#__RefHeading___Toc75597452)

[6.3 Enrollment Visit 40](#__RefHeading___Toc75597453)

[6.4 Product administration 41](#__RefHeading___Toc75597454)

[6.5 Dual-energy X-ray Absorptiometry (DEXA) Scans 43](#__RefHeading___Toc75597455)

[6.6 Concomitant Medications 45](#__RefHeading___Toc75597456)

[6.6.1 Post-exposure Prophylaxis (PEP) 45](#__RefHeading___Toc75597457)

[6.7 Telephone Calls 46](#__RefHeading___Toc75597458)

[6.8 Follow-up Visits 46](#__RefHeading___Toc75597459)

[6.9 Evaluation of Suspected Acute HIV Infection 48](#__RefHeading___Toc75597460)

[6.10 Early Study Product Discontinuation Visit 49](#__RefHeading___Toc75597461)

[6.11 Criteria for Suspension of Study Product 50](#__RefHeading___Toc75597462)

[6.12 Early Study Termination Visit 51](#__RefHeading___Toc75597463)

[6.13 Criteria for Early Study Termination 52](#__RefHeading___Toc75597464)

[6.14 Criteria for Suspension of Study 53](#__RefHeading___Toc75597465)

[6.15 Unblinding 53](#__RefHeading___Toc75597466)

[7 STUDY PROCEDURES FOR PARTICIPANTS DIAGNOSED WITH HIV INFECTION (PART B) 53](#__RefHeading___Toc75597467)

[7.1 Baseline Visit 54](#__RefHeading___Toc75597468)

[7.2 Follow-up Visits 55](#__RefHeading___Toc75597469)

[8 DATA SOURCES 57](#__RefHeading___Toc75597470)

[8.1 Study eligibility assessment 57](#__RefHeading___Toc75597471)

[8.2 Clinical assessment 57](#__RefHeading___Toc75597472)

[8.3 Toxicity monitoring 58](#__RefHeading___Toc75597473)

[8.4 Testing and treatment for sexually transmitted infections (STI) 58](#__RefHeading___Toc75597474)

[8.5 HIV-1 testing 58](#__RefHeading___Toc75597475)

[8.6 Behavioral Assessment 59](#__RefHeading___Toc75597476)

[8.7 Adherence assessments 59](#__RefHeading___Toc75597477)

[8.8 Storage of blood specimens 60](#__RefHeading___Toc75597478)

[9 ADVERSE EVENTS 62](#__RefHeading___Toc75597479)

[9.1 Adverse Events 62](#__RefHeading___Toc75597480)

[9.2 Assessment of Adverse Events 63](#__RefHeading___Toc75597481)

[9.3 Serious Adverse Events 64](#__RefHeading___Toc75597482)

[9.4 Serious Adverse Event Reporting Requirements 66](#__RefHeading___Toc75597483)

[9.5 Clinical Laboratory Abnormalities and other Abnormal Assessments as Adverse Events or Serious Adverse Events 68](#__RefHeading___Toc75597484)

[9.6 Toxicity Management 68](#__RefHeading___Toc75597485)

[10 STATISTICAL CONSIDERATIONS 72](#__RefHeading___Toc75597486)

[10.1 Study Design and Analysis Plan Overview 72](#__RefHeading___Toc75597487)

[10.2 Endpoints 72](#__RefHeading___Toc75597488)

[10.2.1 Primary Endpoints 72](#__RefHeading___Toc75597489)

[10.2.2 Secondary Endpoints 73](#__RefHeading___Toc75597490)

[10.3 Sample Size and Power Calculations 73](#__RefHeading___Toc75597491)

[10.4 Methods of Analysis 76](#__RefHeading___Toc75597492)

[10.5 Safety Analyses for Clinical and Laboratory Measurements 78](#__RefHeading___Toc75597493)

[10.6 Safety Analyses for Behavioral Outcomes 79](#__RefHeading___Toc75597494)

[10.7 Analyses for Study Product Acceptability 80](#__RefHeading___Toc75597495)

[10.8 Independent Safety Review Team 81](#__RefHeading___Toc75597496)

[10.9 Analysis Schedule 82](#__RefHeading___Toc75597497)

[11 RESPONSIBILITIES 82](#__RefHeading___Toc75597498)

[11.1 Investigator responsibilities 82](#__RefHeading___Toc75597499)

[11.2 Sponsor Responsibilities 87](#__RefHeading___Toc75597500)

[11.3 Joint Investigator/Sponsor Responsibilities 88](#__RefHeading___Toc75597501)

[12 ETHICAL CONSIDERATIONS 89](#__RefHeading___Toc75597502)

[12.1 Community Consultation Process 91](#__RefHeading___Toc75597503)

[12.2 Community and Participant Education 92](#__RefHeading___Toc75597504)

[12.3 Informed Consent and Comprehension Test 92](#__RefHeading___Toc75597505)

[12.4 Risk Reduction Counseling 93](#__RefHeading___Toc75597506)

[13 ADMINISTRATIVE STRUCTURE 93](#__RefHeading___Toc75597507)

[13.1 Data Management 93](#__RefHeading___Toc75597508)

[13.2 Case Report Forms (CRFs) 93](#__RefHeading___Toc75597509)

[13.2.1 Paper data 94](#__RefHeading___Toc75597510)

[13.2.2 Electronic data 94](#__RefHeading___Toc75597511)

[13.3 Specimen Collection 94](#__RefHeading___Toc75597512)

[13.4 Study Initiation 94](#__RefHeading___Toc75597513)

[14 REFERENCES 96](#__RefHeading___Toc75597514)

[15 APPENDICES 101](#__RefHeading___Toc75597515)

[Appendix 1 GILEAD INVESTIGATOR’S BROCHURE](#__RefHeading___Toc75597516) 97

[Appendix 2 GILEAD PACKAGE INSERT](#__RefHeading___Toc75597517) 182

[Appendix 3 STUDY SCHEDULES 210](#__RefHeading___Toc75597518)

[Table 1: Study Schedule for HIV-1 Uninfected Participants (Immediate arm) (Part A) 210](#__RefHeading___Toc75597519)

[Table 2: Study Schedule for HIV-1 Uninfected Participants (Delayed arm) (Part A) 211](#__RefHeading___Toc75597520)

[Table 3: Study Schedule for HIV-1 Infected Participants (Part B) 212](#__RefHeading___Toc75597521)

[Appendix 4 FLIP CHART 213](#__RefHeading___Toc75597522)

[Appendix 5 STUDY INFORMED CONSENT 238](#__RefHeading___Toc75597523)

[Appendix 6 COMPREHENSION ASSESSMENT 250](#__RefHeading___Toc75597524)

[Appendix 7 TOXICITY SCALES 255](#__RefHeading___Toc75597525)

[Table 1: NIAID Toxicity Scale 255](#__RefHeading___Toc75597526)

[Table 2: GILEAD Toxicity Scale 269](#__RefHeading___Toc75597527)

[Appendix 8 TOXICITY ALGORITHMS 274](#__RefHeading___Toc75597528)

[Table 1: Management of Clinical and Laboratory Adverse Events (Except Creatinine) 274](#__RefHeading___Toc75597529)

[Table 2: Management of Creatinine Elevations 275](#__RefHeading___Toc75597530)

[Appendix 9 COUNSELING ASSESSMENT 276](#__RefHeading___Toc75597531)

[Appendix 10 CASE REPORT FORMS (CRF) 277](#__RefHeading___Toc75597532)

PROTOCOL SYNOPSIS

| Study Title: | Phase II Extended Safety Study of Tenofovir Disoproxil Fumarate (TDF) for Prevention of HIV among HIV-1 Negative Men |
| --- | --- |
| Study Sites: | AIDS Research Consortium of Atlanta (ARCA), Atlanta, GA  San Francisco Department of Public Health (SFDPH), San Francisco, CA Fenway Community Health (FCH), Boston, MA |
| Objectives: | Primary Objective:  To determine the clinical and behavioral safety and tolerability of oral daily TDF use as pre-exposure prophylaxis (PrEP) to prevent HIV infection in uninfected men.  Secondary Objectives:  To describe the number of HIV infections and to evaluate whether administration of oral TDF affects the genetic characteristics of HIV-1 breakthrough viruses  To assess social and behavioral effects associated with participation in this safety trial  To evaluate participants’ adherence with daily TDF |
| Design: | Phase II, double blind, placebo-controlled, equal (1:1:1:1) randomization |
| Population: | HIV negative men who have sex with men (MSM) |
| Stratification: | By site |
| Sample Size: | 400 HIV negative MSM |

| Duration of Study: | 24 months for HIV negative MSM, 12 months following HIV diagnosis for MSM who become infected while participating in the study |
| --- | --- |
| Randomization  R  A  N  D  O  M  I  Z  E  Daily TDF  Daily Placebo  No treatment for 9 months, then daily TDF  No treatment for 9 months, then daily Placebo  Site B  Site A  Site C |  |
| Enrollment: | Over 24 months |
| Main eligibility criteria: | Healthy, HIV-1 negative MSM who reported anal intercourse in the last 12 months |
| Study procedures/frequency: | Physical examinations, behavioral risk assessments, and laboratory analyses at screening, baseline, months 1, 3 and then every 3 months through 24 months of study. |
| Test product, dose, and mode of administration: | TDF 300 mg po QD. |
| Criteria for Evaluation | Safety  Clinical and laboratory adverse events  Risk behavior change  Adherence to study drug  HIV antiretroviral resistance |
| Statistical Methods | The primary endpoints are to assess the safety and tolerability of TDF as determined by the proportion of participants with grade 3 or 4 abnormalities (clinical adverse events and laboratory toxicities occurring after treatment initiation), the difference in reported risk behavior between the immediate and delayed treatment arms, and overall rates of adherence. Intent-to-treat analysis will be used. |

GLOSSARY OF ABBREVIATIONS AND DEFINITION OF TERMS

| 3TC | lamivudine, Epivir® |
| --- | --- |
| 3TC/AZT | lamivudine/zidovudine, Combivir® |
| ACASI | audio-computer administered interview |
| AE | adverse event |
| AIDS | acquired immunodefienciency syndrome |
| ALT (SGPT) | alanine aminotransferase |
| AST (SGOT) | aspartate aminotransferase |
| ART | antiretroviral therapy |
| BUN | blood urea nitrogen |
| CAB | community advisory board |
| CBC | complete blood count |
| CBO | community based organization |
| CK | creatine kinase |
| CLIA | clinical laboratory improvement amendments |
| CrCl | calculated creatinine clearance |
| CRF | case report form(s) |
| CRO | contract research organization |
| d4T | stavudine, Zerit® |
| DHHS | Department of Health and Human Services |
| ddI | didanosine, Videx® |
| dL | Deciliter |
| DNA | deoxyribonucleic acid |
| EFV | efavirenz, Sustiva® |
| EIA | enzyme-linked immunosorbent assay |
| FDA | (US) Food and Drug Administration |
| FTC | Emtricitabine |
| g | gram(s) |
| GCP | Good Clinical Practice guidelines |
| HAART | highly active antiretroviral therapy |
| HBV | hepatitis B virus |
| HBsAg | hepatitis B surface antigen |
| HDPE | high‑density polyethylene |
| HIV | human immunodeficiency virus |
| HIV‑1 | human immunodeficiency virus type‑1 |
| ICH | International Conference on Harmonisation |
| IRB | Institutional Review Board |
| ITT | intent-to-treat |
| kg | kilogram(s) |
| LLN | lower limit of normal range |
| MedDRA | Medical Dictionary for Regulatory Activities |
| MEMS | Medication Event Monitoring System |
| mEq | milliequivalent(s) |
| mg | milligram(s) |
| mL | milliliter(s) |
| mm | millimeter(s) |
| MSM | men who have sex with men |
| NAT | nucleic acid testing |
| NIAID | National Institute of Allergy and Infectious Diseases |
| NRTI | nucleoside reverse transcriptase inhibitor |
| NNRTI | non-nucleoside reverse transcriptase inhibitor |
| NtRTI | nucleotide reverse transcriptase inhibitor |
| NVP | nevirapine, Viramune® |
| PBMC | peripheral Blood Mononuclear Cells |
| PCR | polymerase chain reaction |
| PI | protease inhibitor |
| PK | Pharmacokinetic |
| PMPA | 9-[(R)-2(phosphonomethoxy)propyl]adenine monohydrate (tenofovir) |
| PMPA prodrug | 9-[(R)-2-[[bis[[(isopropoxycarbonyl)oxy]methoxy] phosphinyl]-methoxy]propyl]adenine fumarate (1:1) (tenofovir disoproxil fumarate, tenofovir DF, TDF) |
| PEP | post-exposure prophylaxis |
| PrEP | pre-exposure prophylaxis |
| QD | once daily |
| RNA | ribonucleic acid |
| RT | reverse transcriptase |
| SAE | serious adverse event |
| STI | sexually transmitted infection |
| TDF | tenofovir DF, Viread® |
| UA | Urinalysis |
| ULN | upper limit of normal |

# INTRODUCTION

## Background

It has been estimated that 14,000 persons become infected with the human immunodeficiency virus type one (HIV-1) daily, and approximately 40 million individuals are currently infected worldwide (1). As of January 2003, 859,000 AIDS cases had been reported to the Centers for Disease Control and Prevention (CDC) in the United States, and an estimated 143,904 people are now living with HIV infection (2). Of the approximately 35,000 new infections identified annually in the United States, 32% are among men who report sex with men (MSM) and 17% are acquired through heterosexual sex (2). Although, substantial efforts have been devoted toward microbicide and vaccine development, no biomedical product effective at preventing HIV infection has yet been identified. Current efforts among high risk populations have focused on behavioral strategies, encouraging the reduction in the number of sexual partners, monogamy, and consistent and correct condom use for the prevention of sexually transmitted HIV. However, another effective, easy to use method of prevention is urgently needed.

Current animal and human study data suggest that antiretroviral agents used to treat HIV-1 infection may be efficacious when used following HIV exposure (3-10) and animal data suggest that antiretroviral agents may be efficacious pre-exposure as well (7-9). Recently, a non-nucleoside reverse transcriptase inhibitor (NNRTI), nevirapine, was tested in a small phase I/II clinical trial (3) as a potential chemoprophylaxis agent. Although it was generally well-tolerated, there was evidence of hepatotoxicity, and additional clinical reports suggest that nevirapine might be ill suited for pre-exposure prophylaxis (PrEP) because of its unacceptable frequency of side effects and toxicity, especially among HIV uninfected individuals, (11-15) and the ease in which resistance develops (11-14). Another recently approved antiretroviral, Viread® (Tenofovir disoproxil fumarate [TDF]), a nucleotide reverse transcriptase inhibitor (NtRTI), has a lower toxicity profile and may be a more promising PrEP agent.

The purpose of this study is to assess the clinical and behavioral safety of TDF among HIV-1 negative men for its potential further investigation as a chemoprophylactic agent in the prevention of HIV infection as determined by the proportion of participants in each arm who experience adverse events or report different rates of HIV risk behaviors.

## TDF

### General Information

Tenofovir (9‑[(R)‑2‑(phosphonomethoxy)propyl]adenine monohydrate, PMPA) is an adenine nucleoside monophosphate (nucleotide) analog with activity against retroviruses, including HIV‑1 and HIV‑2 (16,17). Tenofovir is metabolized intracellularly to tenofovir diphosphate (PMPApp), which is a competitive inhibitor of HIV‑1 reverse transcriptase (RT) that terminates the growing DNA chain. Due to the presence of a phosphonate group, tenofovir is negatively charged at neutral pH, thus limiting its oral bioavailability. Therefore, tenofovir disoproxil fumarate (TDF, Viread®, (9-[(R)-2-[[bis[[(isopropoxycarbonyl)oxy]‑ methoxy]phosphinyl] methoxy]propyl]adenine fumarate 1:1) a prodrug of tenofovir was developed. TDF is orally bioavailable in animals and humans and is rapidly converted to tenofovir on absorption (18). Further information regarding TDF, its toxicology, efficacy and safety profile will be reviewed in the below text and is available in the Gilead Investigator’s Brochure (Appendix 1) and Viread® Package Insert (Appendix 2).

### Preclinical Pharmacology and Toxicology

TDF is a nucleotide reverse transcriptase inhibitor (NtRTI) and as such is distinct from nucleoside reverse transcriptase inhibitors (NRTIs). Unlike nucleosides, tenofovir undergoes only two rather than three consecutive phosphorylation steps to its active metabolite PMPApp.

The non-clinical toxicology of TDF has been studied in mice, rats, rabbits, dogs, and rhesus macaques. The principal organs of toxicity following TDF administration were the gastrointestinal tract (rats), kidney, and bone. Nephrotoxicity was the dose-limiting toxicity associated with TDF administration in dogs and monkeys. Reductions of serum phosphate were noted in rats, dogs, and monkeys, and chronic high-dose TDF administration to rats and dogs demonstrated some loss of bone mineral density; this loss seemed to be nonprogressive or minimally progressive between weeks 13 and 42. It is suspected that this osteopenia may be related to accelerated bone resorption secondary to reduction of the intestinal phosphate absorption and/or mild renal phosphate leak that develops with chronic treatment. Osteomalacia was documented in rhesus macaques administered chronic daily administered injections 25x the strength of human doses (Appendix 1).

No adverse effects were detected in studies examining the effects on fertility and early embryonic development in rats or rabbits. TDF was not mutagenic in the in vitro *Salmonella*/*Escherichia coli* mammalian microsome reverse assay with the exception of a minimally positive response seen in one of the studies, which was considered to be equivocal (Appendix 1).

### Clinical Trials

The US Food and Drug Administration (FDA) approved Viread® (TDF) in October 2001 for use in combination with other antiretroviral therapeutic (ART) agents for the treatment of HIV‑1 infection. Over 12,000 patients have participated in clinical trials or expanded access studies involving TDF, and TDF has been shown to be generally safe, effective, and well tolerated. (Appendix 2). The TDF clinical trials program includes seven completed phase I trials (studies 701 [intravenous tenofovir], 909, 914, 917, 919, 930, and 932), one phase I/II trial (study 901), one phase II trial (study 902) and three phase III trials (studies 907, 908, 910) (Appendix 1).

Ongoing clinical studies are examining TDF for use in new populations or for new indications. Three studies are being conducted among HIV-infected pediatric populations to examine a suspension formulation for safety and pharmacokinetics. Other studies are assessing safety when co-administered with adefovir dipivoxil for the treatment of lamivudine-resistant hepatitis B virus (HBV) infection among adult patients co-infected with HIV and HBV and as an intravaginal tenofovir topical gel formulation among HIV infected and uninfected women.

In addition, two ongoing phase III trials, 903 and 934, are being conducted among HIV-1 infected adult participants. Study 903 is a randomized, double-blind three-year trial designed to assess the efficacy and safety of TDF vs. stavudine (d4T) with background therapy of lamivudine (3TC) and efavirenz (EFV). Study 934, initiated in August 2003, is an open-label, multicenter clinical trial that will compare TDF, emtricitabine, and EFV with the combination tablet (lamivudine/zidovudine) and EFV.

### Pharmacokinetics

The pharmacokinetics (PK) of TDF has been specifically evaluated in seven trials (studies 901, 907, 909, 914, 919, 930, and 932). These studies demonstrated that the bioavailability of TDF is enhanced by administration with food, TDF is eliminated by both active tubular secretion and glomerular filtration, and the TDF serum half‑life of ~17 hours and intracellular half-life of 10-50 hours supports a once-daily dosing schedule. Following oral administration of 300mg of TDF to HIV-infected patients in the fasted state, maximum serum concentrations are achieved in 1.0 + .4 hours. Since TDF is eliminated by the kidneys, a dose adjustment is necessary for patients with significant renal impairment. The only significant drug interaction reported is with didanosine (Videx® EC, [ddI]) in which ddI systemic exposures increased when administered simultaneously with TDF (Appendix 2).

### Safety

Numerous clinical studies (901, 917, 902, 903, 907, 908, and 910) have demonstrated TDF to have an excellent safety profile (Appendix 1). Assessment of adverse reactions has been primarily based on four studies among HIV-infected persons (902, 903, 907, and 910) in which treatment-experienced and naïve patients received TDF or placebo as part of their ART regimens for at least 24 weeks. Adverse event rates were comparable between the TDF and placebo groups. In the TDF arms, the most common adverse events were mild to moderate gastrointestinal events, i.e., nausea, diarrhea, vomiting and flatulence, occurring in 3-11% of treatment-experienced patients. In studies 907 and 903, rates of creatinine elevation and hypophosphatemia (serum phosphorus <2.2 mg/dl) were similar between the TDF and placebo groups. Among the TDF study patients, 3-4% experienced a serum creatinine increase of at least 0.5 mg/dL from their baseline level; these increases were generally transient and only two persons exceeded 2.1 mg/dL. Hypophosphatemia was detected in 6-16% of TDF study patients (Investigator’s Brochure). In study 902, none of the patients discontinued the study because of TDF-related serum creatinine elevations or hypophosphatemia (19,20).

In the first 48 weeks of the ongoing phase III study (903) comparing TDF to stavudine (d4T) as the third component of ART, grade 3/4 abnormalities were detected in 29% of persons in the TDF group. Elevations in creatinine kinase (8%) and amylase (7%) were the most frequently reported grade 3/4 laboratory abnormalities. Only 3 patients discontinued the study due to laboratory toxicities. Four participants had serious adverse events (anemia, peripheral neuritis, bronchitis, and gynecomastia) that were considered to be possibly related to study drugs. There were no bone fractures attributed to vertebral compression, minimal trauma, or otherwise considered to be related to study drug. The table below lists the grade 2-4 adverse events reported by at least 3% of TDF recipients in study 903 compared to the d4T arm.

**Grade 2-4 Adverse Events Reported in > 3% of TDF Recipients in Study 903 (0-48 weeks)**

| **Type of Event** | **TDF+3TC+EFV**  **(N=299)** | **D4T+3TC+EFV**  **(N=301)** |
| --- | --- | --- |
| **General** |  |  |
| Headache | 10 % | 11% |
| Pain | 7 % | 6 % |
| Fever | 5 % | 6% |
| Abdominal pain | 4 % | 8 % |
| Back pain | 4% | 3% |
| Asthenia | 3% | 5% |
| **Gastrointestinal** |  |  |
| Nausea | 5% | 6 % |
| Diarrhea | 6% | 6% |
| Vomiting | 3% | 6% |
| Dyspepsia | 3% | 2% |
| **Neurologic** |  |  |
| Depression | 7% | 5% |
| Insomnia | 4% | 6% |
| Abnormal dreams | 3% | 3% |
| Dizziness | 3% | 5% |
| **Respiratory** |  |  |
| Pneumonia | 3% | 3% |
| **Dermatologic** |  |  |
| Rash (includes pruritus, urticaria) | 15 % | 11 % |

At 48 and 96 weeks, 1% and 3% of TDF patients experienced a serum creatinine increase of at least 0.5mg/dl from their baseline level and 5% and 6% experienced hypophosphatemia, respectively (Appendix 1 and indicated in tables below).

**Incidence of Creatinine Elevations Detected in Study 903 Participants (0-96 weeks)**

| **Serum Creatinine** | **TDF+3TC+EFV**  **(N=299)** | **D4T+3TC+EFV**  **(N=301)** |
| --- | --- | --- |
| Grade 1 (> 0.5 from baseline) | 3 % | 2% |
| Grade 2 (2.1-3.0) | <1 % | 0 |
| Grade 3 (3.1-6.0) | 0 | <1% |
| Grade 4 (<1.0) | 0 | 0 |

**Incidence of Hypophosphatemia Detected in Study 903 Participants (0-96 weeks)**

| **Hypophosphatemia** | **TDF+3TC+EFV**  **(N=299)** | **D4T+3TC+EFV**  **(N=301)** |
| --- | --- | --- |
| Grade 1 (2.0-<2.2) | 3 % | 3% |
| Grade 2 (1.5-1.9) | 3 % | 2% |
| Grade 3 (1.0-1.4) | <1 % | <1% |
| Grade 4 (<1.0) | 0 | 0 |

Whole-body dual-energy x-ray absorptiometry (DEXA) scans among study 903 participants demonstrated greater percent decreases in bone mineral density (BMD) from baseline in the TDF arm (spine, -3.3% + 3.9; hip -3.2% + 3.6) compared to the D4T arm (spine, -2.0% + 3.5; hip -1.8% + 3.3). At 48 weeks 6% of 300 TDF arm participants and 2% of 300 stavudine participants met the definition for a decrease in BMD and 13% and 10% at 96 weeks, respectively. However, 25% of participants were already osteopenic at baseline and this data did not correlate with incidence of fractures (11 in the stavudine and 4 in the TDF arm) through 3 years of the study. Additionally, all fractures reported were traumatic, except for one in the stavudine arm which was a spontaneous compression fracture. Thus, the clinical significance of these decreases is unknown (Appendix 1) (21).

The study 903 toxicity results are comparable to the phase III study (910) in which long‑term follow up showed a low rate of adverse events or laboratory abnormalities leading to TDF discontinuation despite a mean patient treatment duration of greater than two years, and as long as four years in others.

Several small brief clinical studies have been performed among HIV uninfected persons (studies 909, 914, 919, 929, 930, 932, 939, 943, and 984). The studies among healthy persons were primarily conducted to examine the pharmacokinetics (PK) of tenofovir alone or in combination with other ART. They enrolled small numbers of participants and were conducted for a median of 24 days (range 10-99 days); none examined extended safety. Data pooled from two small trials including HIV‑infected persons (n = 17, studies 901 and 907) and healthy volunteers (n = 36, study 914) demonstrated no significant differences in tenofovir PK by HIV infection status, gender, age (age range 19 to 57 years) or body weight (range, 50 to 112 kg). Other studies confirmed the safety of TDF among persons who used methadone, oral anticontraceptives, or had impaired hepatic function, and established dosage guidelines for patients with moderate to severe renal impairment (Appendix 1).

### Efficacy

Several large randomized clinical trials among HIV-infected persons have demonstrated TDF to be effective at reducing plasma HIV-1 RNA concentrations (studies 907, 902) and comparable to other ART regimens (903) (21). TDF is active against some HIV-1 isolates that are resistant to zidovudine, didanosine, abacavir, and lamivudine, and against multinucleoside drug-resistant variants carrying the Q151M mutation within the reverse transcriptase. Virus susceptibility to TDF is enhanced with the presence of the M184V mutation (22,23). TDF selects for a K65R mutation in the reverse transcriptase along with some other antireretrovirals, e.g. abacavir, didanosine. The K65R mutation is associated with a 3-4 fold reduction in tenofovir susceptibility in vitro and reduces susceptibility to lamivudine (22,24,25). Fortunately, it appears this signature mutation develops infrequently. Studies of TDF-containing regimens in HIV-infected persons for up to 96 weeks demonstrated infrequent development of K65R (2-3%; the median time to development of the K65R mutation was 27 weeks) and many of these persons were taking other antiretrovirals that could have contributed to development of this mutation(21,25,26).

Given its strong potency and favorable resistance profile, two recent studies yielded surprising results of poor efficacy when TDF was used as part of a triple nucleoside combination (27,28). Both studies noted high rates of virologic non-response, 58% and 49%, respectively, among patients receiving TDF in combination abacavir and lamivudine. Genotypes from the non-responders’ isolates yielded a lone M184V mutation, the M184V + K65R mutation, or wild-type virus; no patient failed with an isolated K65R mutation. It is uncertain what contributed to the non-virologic response. Hypotheses include intracellular pharmacologic interactions, or low genetic resistance barrier to the drug combination.

### Clinical Experience

Over 153,000 persons have now received TDF in a clinical setting, and relatively few adverse events have been reported. Those reported include renal impairment, nausea, rash and weakness (29-34). Renal adverse events have been the most extensively documented. A number of cases of tenofovir-associated nephrotoxicity involving renal failure, proteinuria, renal tubular dysfunction with Fanconi’s syndrome, and diabetes insipidus have been described among patients using TDF (31-37). In many of these cases, possible contributing factors included other ART, underlying renal disease, and low body weight.

TDF’s potential for mitochondrial toxicity and the associated lactic acidosis as compared with other NRTIs appears to be low (38,39). Two case reports noted fatal lactic acidosis in patients using TDF (30,40), however both patients were also receiving ddI, and one patient had multiple medical problems (30). Elevations of creatine phosphokinase (CPK) have been seen in patients using TDF, however these elevations may be due to concomitant use of acyclovir and/or atorvastatin (29). Given the concomitant use of other medications, in many clinical situations, it is difficult to assess the role of TDF in contributing to the toxicity (30-32,34).

In general, clinical experience suggests that oral, once-daily TDF appears comparable to placebo for safety, has durable efficacy in HIV-1 naïve and treatment-experienced patients and is generally safe when used in combination with other antiretrovirals in HIV-infected persons. In addition, other small studies suggest that TDF may lower lipid levels (41). However, the long-term safety profile for healthy, HIV uninfected persons has not been established.

### Non-Human Primate Challenge Studies

Tenofovir has demonstrated potent antiviral activity in several challenge experiments conducted among non-human primates providing strong support for both the pre-exposure as well as post-exposure use of TDF in the prevention of retroviral infections. These animal studies investigated TDF’s potential protective effect in retroviral challenges involving intravenous, oral, and intravaginal routes of transmission.

In one of the landmark studies, researchers demonstrated that tenofovir prevented simian immunodeficiency virus (SIV) infection in rhesus macaques administered once daily tenofovir following SIV intravenous challenge. Treatment was initiated either 48 hours before, 4 hours after, or 24 hours after SIV inoculation and continued for up to four weeks. All animals administered tenofovir remained healthy and free of SIV, whereas control macaques became infected and died quickly (7). A second study also demonstrated protection from simultaneous oral SIV and intravenous simian/human immunodeficiency virus (SHIV) inoculations in newborn macaques administered a two-week course of tenofovir starting immediately after viral inoculation (42).

Subsequent studies noted that both the timing of treatment initiation and the duration of therapy appeared to be important for protection. Two studies demonstrated that 2 doses of tenofovir administered 4 hour before and 20 hours after oral SIV inoculation protected newborn macaques (8) and suggested that partial protection could be achieved with lower and fewer doses (9). However, other studies demonstrated that although protection was achieved with a 28-day course of tenofovir started 12, 24, or 36 hours after retroviral exposure (intravaginal or intravenous), efficacy was reduced if therapy was initiated at 48 or 72 hours post inoculation or with shorter durations of treatment (10,43). Thus, although the optimal timing of dosing and duration of therapy are unknown, this promising data in animal models merits further investigation of tenofovir as a potential agent in the prevention of HIV infection.

## Behavioral Disinhibition

The possibility that this and other biomedical HIV prevention interventions might adversely influence HIV risk behaviors and/are undermine behavioral risk reduction strategies are issues of concern (44). Persons may feel falsely protected while using such an intervention. As a result, there may be increased HIV risk behaviors, potentially increasing HIV and other sexually transmitted infections (STIs).

HIV vaccine modeling studies suggest that even in the setting of extremely effective HIV vaccines, failure to simultaneously implement efficacious HIV risk behavior interventions will make prevention of HIV extremely difficult (45). Balancing these concerns of behavioral disinhibition are data from an actual phase III HIV vaccine efficacy trial indicating that HIV risk behavior initially declined and then remained at baseline or below for the majority of the participants (Bartholow B., in press). Similarly, a feasibility study of non-occupational post exposure prophylaxis among predominantly San Francisco MSM with high-risk sexual and drug-use exposures demonstrated a significant overall reduction in high-risk sex acts (46).

It is critical to evaluate HIV risk behavior and integrate behavioral risk reduction strategies within the context of HIV biomedical intervention studies so that adverse behavioral manifestations may be prevented among current or future participants in research and/or HIV prevention programs. If new biomedical technologies are found efficacious, it will also be important to conduct studies that address use, acceptability, and risk-behavior in “real world” settings. In addition, it will be essential to evaluate existing and novel behavioral risk reduction strategies used in tandem with any new biomedical prevention method.

## Rationale for the Current Study

Current efforts among high risk populations have focused on behavioral strategies, encouraging the reduction in the number of sexual partners, monogamy, and consistent and correct condom use for the prevention of sexually transmitted HIV. However, despite many years of designing, testing, and implementing behavioral interventions in the United States, the rate of new infections in the US has remained elevated for the last ten years (47,48). It is clear in order to reduce the incidence of HIV, both effective biomedical and behavioral prevention strategies are needed.

TDF has been selected for investigation as PrEP agent because of its unique pharmacologic characteristics (single daily tablet, low incidence of side effects, striking anti-HIV potency, and favorable resistance profile) and encouraging primate data. Two large phase IIb studies are scheduled for implementation by Family Health International (FHI) and University of California- San Francisco (UCSF) among 2000 high-risk women in four countries (Cambodia, Ghana, Nigeria, and Cameroon) to demonstrate the safety and effectiveness of once daily oral TDF for the prevention of HIV infection (49,50). Another potential study phase IIb may occur among men in Malawi. A CDC phase II study will provide extended clinical and behavioral safety data for men who have sex with men (MSM) who are at risk of acquiring HIV infection. TDF tablets and placebo will be provided by the pharmaceutical study sponsor, Gilead Sciences. The phase II clinical trial will be conducted in compliance with the protocol, Good Clinical Practices (ICH/GCP), and the applicable regulatory requirements of the US.

The CDC study will assess both the extended clinical and behavioral safety issues among MSM. Clinical safety will be evaluated by the use of standard clinical trial adverse event reporting. Although TDF has an excellent safety profile, two body organ systems (renal and bone) may exhibit signs of long-term toxicity and will receive close clinical and laboratory monitoring and assessment during this study. Behavioral safety will be assessed through participant reporting of HIV risk behavior and perceptions of treatment assignment and TDF efficacy using self-administered computerized questionnaires and testing for sexually transmitted infections (STIs). Participants will be randomized to one of four study arms. Two arms will receive daily oral TDF or placebo for 24 months (immediate treatment). The other two arms will enroll participants who receive no study product for the first nine months and then will receive either TDF or placebo for the remaining 15 months of the study (delayed treatment). The presence of the immediate and delayed treatment arms will allow for comparison of HIV risk behavior between those receiving study product and those not receiving study product. All participants will receive regular HIV counseling and testing by trained counselors.

# STUDY OBJECTIVES

Purpose: To evaluate the use of oral daily TDF by MSM at risk for HIV infection to determine its potential suitability for further evaluation as a preventive intervention

Primary Objectives

To evaluate the extended safety and tolerability of daily TDF 300 mg use by HIV negative MSM through

1. clinical and laboratory adverse events
2. self-reported risk behavior

Secondary Objectives

1. To describe the incidence of HIV infections and to evaluate whether administration of oral TDF affects the genetic characteristic of HIV-1 breakthrough viruses
2. To evaluate participants’ adherence with daily TDF use
3. To evaluate social and behavioral effects associated with participation in this safety trial

# STUDY DESIGN

This protocol describes a randomized, double-blind, placebo-controlled three-site study to evaluate the safety and tolerability of daily oral TDF among HIV-1 negative MSM.

Four hundred healthy, HIV‑1‑negative MSM will be randomized to one of four treatment arms:

Group 1: TDF 300 mg po QD

Group 2: Placebo po QD

Group 3: No study product for 9 months, followed by TDF 300 mg po QD

Group 4: No study product for 9 months, followed by placebo po QD

Groups 1 and 2 will be referred to as the immediate treatment arms and will receive study product for the entire 24 months of the study. Groups 3 and 4 will be referred to as the delayed treatment arms, and will receive study product starting at 9 months and ending at 24 months, for a total duration of 15 months on study product. For the purposes of this study, a month is considered 30 days. Participants will be stratified by enrollment site.

Participants will be monitored for clinical safety using periodic physical examinations, adverse event reporting, and serial laboratory testing. Given the uncertainty regarding the clinical significance of whole-body duel-energy x-ray absorptiometry [DEXA] findings and their lack of correlation with other clinical data (e.g. fractures), SFDPH site participants will receive DEXA scans at screening and two follow-up visits (at 12 and 24 months in the immediate arm, at 9 and 24 months in the delayed arm), and at the time of study discontinuation, if at least 6 months has passed since the previous scan. Behavioral safety will be primarily assessed by comparing reported risk behaviors between the immediate (pill-groups 1 and 2) and delayed (no pill-groups 3 and 4) treatment arms and will be evaluated by the use of periodic risk behavior assessments via audio computer-assisted self interview (ACASI) and regular laboratory testing for HIV and sexually transmitted infections (STI) including syphilis, *Neisseria gonorrhoeae* (GC), and Chlamydia trachomatis (CT). Additionally, measurements of adherence will be evaluated to determine product acceptability.

Study Schema

R

A

N

D

O

M

I

Z

E

Daily TDF

Daily Placebo

No treatment for 9 months, then daily TDF

No treatment for 9 months, then daily Placebo

Site B

Site A

Site C

Anticipated Timeline

Months

|  | 0-6 | | 7-12 | | | | 13-18 | | | 19-24 | | 25-30 | 31-36 | | 37-42 | |
| --- | --- | --- | --- | --- | --- | --- | --- | --- | --- | --- | --- | --- | --- | --- | --- | --- |
| Screening/Enrollment |  | | | |  | |  | | |  | |  |  | |  | |
| On Study |  | | | | | | | | | | | | |  | | |
| Safety Reviews |  |  | |  | |  | |  |  | |  |  |  | |  | |
| Closeout |  | |  | | | |  | | |  | |  |  |  | |  |
| Analyses |  | |  | | | |  | | |  | |  |  |  | | |

# PARTICIPANTS

## Target Populations

To assess the extended safety of TDF among HIV negative MSM, study sites need to have access to male populations with definable and predictable risks of HIV-1 infection based on established seroincidence rates. The recently completed AIDSVAX B/B HIV vaccine phase III efficacy trial included MSM enrolled from 56 sites in the United States, and HIV seroincidence was 2.8% (51). Since high-risk women will be exclusively studied in the FHI and UCSF studies, they will not be included in this study.

## Number of Participants and Participant Selection

A total of 400 HIV-1 uninfected, healthy MSM are planned for enrollment in this study. Men who are not known to be HIV-1 infected but who are at-risk of HIV-1 infection through sexual activity will be screened for possible trial participation. Participants may be enrolled from pre-existing cohorts or may be newly recruited for this study.

In the screening period, all potential participants will have a brief medical history and a directed physical exam to ensure that they are healthy and meet all eligibility criteria, and to document all past and baseline chronic, clinically significant medical conditions that may be relevant to the interpretation of adverse events during the study. Eligible subjects will be randomized to one of 4 study arms 1) immediate daily TDF (300 mg) 2) immediate daily placebo 3) no study product for 9 months followed by daily TDF for the remainder of the study; or 4) no study product for 9 months followed by daily placebo for the remainder of the study. Those participants who prematurely discontinue study drugs will be asked to continue with the scheduled study visits and will not be replaced.

## Inclusion Criteria

Participants must meet all of the following inclusion criteria within 45 days prior to randomization, unless specified otherwise, to be eligible for study entry:

*General*

- Healthy biologic male (male at birth)
- 18-60 years of age
- HIV-1 negative by licensed, commercially available, FDA-approved whole blood rapid enzyme immunoassay (EIA) at screening and enrollment
- Reports any anal sex with a man in the last 12 months
- Able to understand and pass comprehension assessment questionnaire
- Able to understand and sign a written informed consent form, which must be obtained prior to initiation of study procedures
- Able to understand English

*Biologic criteria*

- Adequate renal function:
- Calculated creatinine clearance (CrCl) ≥ 70 mL/min according to the Cockcroft‑Gault formula:

Male: (140 - age in years) x (wt in kg) = CrCl (mL/min)
 72 x (serum creatinine in mg/dL)

- Hepatic transaminases (AST and ALT) ≤  2x upper limit of normal (ULN)
- Total bilirubin ≤ 1.5 mg/dL
- Adequate hematologic function: (absolute neutrophil count ≥ 1,500/mm^3^; platelets ≥ 100,000/mm3; hemoglobin ≥ 9.5 g/dL)[[1]](#footnote-2)
- Serum amylase ≤ 1.5 x ULN
- Adequate biochemical profile: within normal limits for serum phosphorus, potassium, sodium, and calcium.
- Hepatitis B surface antigen negative
- Normal urine dipstick or urinalysis (UA)
- Negative glucose
- Negative or trace protein, and
- Negative hemoglobin

## Exclusion Criteria

Participants who meet any of the following exclusion criteria are not eligible for the study.

- Active untreated syphilis
- Current uncontrolled hypertension (blood pressure > 160/100 mmHg)
- Mutually monogamous (members of couple engage in sexual activities only with each other, excluding all others) relationship for > one year with a known HIV antibody negative partner
- History of chronic renal disease, known osteoporosis, osteomalacia, or osteopenia
- Bone mineral density Z score < -2.5 at the total spine, total hip or femoral neck on screening (done at SFDPH only), or current treatment for secondary causes of low bone mineral density.
- Current or expected participation in other longitudinal HIV behavioral or biomedical research study
- Current HIV antiretroviral use
- Receiving or planning to receive on-going therapy with any of the following:
- Nephrotoxic agents
- aminoglycoside antibiotics
- intravenous amphotericin B
- cidofovir
- cisplatin
- foscarnet
- intravenous pentamidine
- other agents with significant nephrotoxic potential
- Probenecid
- Metformin
- Experimental or investigational agents
- Previous or expected requirements for the administration of immunosuppressive/ immunomodulatory therapy (e.g. chronic systemic corticosteroids, interferon, interleukins, chemotherapy, radiation).
- Evidence of a gastrointestinal malabsorption syndrome or chronic nausea or vomiting which may confer an inability to receive an orally administered medication.
- Current alcohol or substance abuse judged by the investigator to potentially interfere with participant compliance.
- Imminently life-threatening medical conditions (malignancy, immunosuppressive disease [e.g. lymphoma]), or other serious disease or conditions (e.g. cardiovascular, renal, diabetes) within the last 5 years or that are unstable and/or require chronic medication that would, in the investigator’s judgment, impede compliance with study requirements and complicate the interpretation of adverse events
- Expected to be non-compliant with study visits or planning to move within 24 months to an area where the study will not be conducted
- Any other clinical or social condition, prior therapy, occupation, or other responsibility, that, in the opinion of the investigator, would interfere with, or serve as a contraindication to study participation or compliance with the dosing requirements.

# TREATMENT PLAN AND STUDY PRODUCT

## Treatment Plan

Participants will be assigned a participant number at the time of screening consent. Once eligibility has been confirmed, the site will be given a specific numeric identifier that corresponds to the product assignment. All screening and baseline tests and procedures must be completed prior to the receipt of the first dose of study product. Initiation of treatment with study product must take place within 24 hours after product dispensation.

## Randomization

Participants will be randomly assigned to one of the four arms: immediate study product receipt (TDF or placebo) or delayed study product receipt (TDF or placebo) in a 1:1:1:1 ratio. A CDC statistician will develop the allocation sequence using a permuted blocks randomization scheme. In a block of 8 study numbers, 2 assignments will be to immediate TDF, 2 to immediate placebo, 2 to delayed TDF, and 2 to delayed placebo. Randomization codes will be provided to Gilead Sciences, Inc., and study bottles will be labeled and filled with TDF or placebo using these codes and shipped to the appropriate site. Participants will be randomized to a group only after they have consented to participate and they have met the eligibility requirements for the study. Randomization will be stratified by study site. The list of treatment assignments and the assignment codes will be known only to the statistician and designated individual at Gilead Sciences, Inc., and only CDC will have access to the list of treatment assignments should the need for this information arise.

Group assignments will be concealed in sequentially numbered, sealed envelopes that will be distributed to each site by CDC and maintained in a secure location. After a participant has been properly enrolled in the study, a designated study staff member will obtain the next randomization envelope in sequence and be provided the information indicating the participant’s assignment to a delayed or immediate arm and the randomization number (bottle identification number) assigned.

### Blinding

TDF and placebo will be provided to the study sites in identical bottles labeled only with the participant ID number. If HIV testing reveals confirmed HIV-1 infection, infected participants will be informed about their infection and asked to permanently discontinue study product, and continue with Part B of the study. All HIV-infected participants will receive HIV genotypic and phenotypic resistance testing at diagnosis, and they (and their health care providers if they so request) will receive these results. Since unblinding is not necessary for future clinical management, the participant, study site, and staff will remain blinded as to their treatment assignment group until the study is closed and the database locked.

CDC will be notified of all serious adverse events. Unblinding will occur only in urgent situations where the need for the knowledge of product receipt is shown to be critical to the participant’s care. The unblinding request with the rationale for the request should be provided to CDC and the medical monitor.

### Minimizing unblinding related to adverse events

The study design requires double-blind administration of study product (TDF or placebo). In previous studies of TDF, there were no adverse events exclusively associated with the active study or that required knowledge of the product assignment to administer care. Thus, the observation of any particular adverse event should not require unblinding of either researcher or participant.

### Minimizing potential for self-unblinding

Considerable bias could be introduced if participants were unblinded and risk behavior changed differentially in the treatment groups. For example, if participants have their blood or study product tested outside of the study, they may be able to determine if they received TDF or placebo. If TDF recipients assumed that they were protected and increased their risk-taking behavior while placebo recipients’ risk behavior was unchanged, the resulting increase in infections among TDF recipients could offset any true protection offered by TDF and impede the ability to accurately assess safety.

To minimize the risk of bias related to ascertainment of product assignment, the following actions will be taken. First, all participants will be counseled not to assume any protection from study product and to avoid high risk behaviors. Second, at enrollment, 6 month intervals, and study conclusion, participants will be asked about their perceived treatment assignment. Third, at enrollment and at subsequent 3 month intervals during the study, participants will self-report their risk-taking behavior by ACASI and receive HIV testing and counseling regarding risk behavior reduction. The information collected from the risk assessments will be reviewed at each safety review and at the conclusion of the study to assess the comparability of self-reported risk behaviors for the initial 9 months between the delayed and immediate treatment arms and before and after treatment initiation for the delayed treatment arms.

## Study Product

TDF tablets are 300mg, light blue, almond‑shaped, film‑coated tablets for oral administration. Each tablet contains the following inactive ingredients: microcrystalline cellulose, lactose monohydrate, pregelatinized starch, croscarmellose sodium, and magnesium stearate. The film coating consists of lactose monohydrate, hydroxypropyl methylcellulose, titanium dioxide, triacetin, and FD&C blue #2 aluminum lake for color. Placebo tablets have an identical appearance to the TDF tablets and contain the inactive ingredients listed above and denatonium benzoate to provide a bitter taste to match the active tablets (Appendix 2). Gilead Sciences, Inc., will supply TDF and placebo tablets.

### Study Product Handling and Packaging

TDF or placebo tablets will be packaged at the Gilead manufacturing facility in white high-density polyethylene (HDPE) bottles. For the immediate arms, bottles will be packaged in kits of 7 bottles per participant. For the delayed arms, bottles will be packaged in 2 kits of 7 and one kit of 4 per participant. Each bottle will contain 30 tablets and a single silica gel canister to protect the product from humidity and fiber packing during handling and shipping. Study product should be stored before dispensing to participants at 25°C; excursions permitted to 15-30°C. Each bottle will be labeled with an identification number, the protocol number, administration instructions, expiration date, lot number, and sponsor address. Additional information will be included according to the protocol requirements and local law.

At the study sites, a designated staff person will transfer study product into another white HDPE fitted with a Medication Event Monitoring System (MEMS®) cap (52). Each bottle with the MEMS cap will be labeled with an identification number, the protocol number, administration instructions, expiration date, lot number, and sponsor address. Study product will be dispensed at enrollment, months 1 and 3 and then every 3 months thereafter for participants randomized to the immediate treatment arms. Participants randomized the delayed treatment arms will receive study product at 9, 10 and 12 months and then every 3 months thereafter. Participants will be instructed to return unused study product at each study visit. The investigator will be responsible for maintaining accurate records for all study bottles dispensed and returned. The inventory must be available for inspection by the study monitor. Study product supplies, including partially used or empty bottles and the dispensing logs, must be accounted for by the study monitor prior to destruction or return.

## Excluded Concomitant Medications

The following medications are excluded while participating in the study:

- Nephrotoxic agents
  - aminoglycoside antibiotics
  - intravenous amphotericin B
  - cidofovir
  - cisplatin
  - foscarnet
  - intravenous pentamidine
  - other agents with significant nephrotoxic potential
- Probenecid
- Metformin
- Systemic chemotherapeutic agents (i.e., cancer treatment medications)
- Chronic systemic corticosteroids
- Interleukin‑2 (IL‑2)
- Other immunosuppressive/immunomodulatory agents
- Experimental or investigational agents
- Antiretrovirals (other than PEP, see 6.6.1)
- Radiation therapy

Should participants need to start treatment with any excluded concomitant medication, study product will be discontinued. In instances where an excluded medication is initiated prior to study product discontinuation, the investigator must notify CDC and the medical monitor as soon as they are aware of the use of the excluded medication.

# MAIN STUDY PROCEDURES (PART A)

The study procedures to be conducted for each participant enrolled in the main study (Part A) are presented in Tables 1 and 2 (Appendix 3) and detailed in the text that follows. Participants who become HIV-I infected during the course of the study will be asked to continue under a separate visit schedule, referred to as Part B, presented in Table 3 (Appendix 3) that will be covered in Section 7. All routine laboratory specimens will be sent to local institutional laboratories for analysis. Other laboratory specimens will be sent to a designated central lab for analysis or storage.

Any deviation from protocol procedures should be noted in the Case Report Forms (CRFs) or the participant file and the CDC or Contract Research Organization (CRO) should be notified depending on the nature of the deviation.

## Recruitment

Participants will be followed for 24 months. Recruitment will be done using a variety of previously approved and successful recruitment techniques, e.g. outreach, the internet, referrals, etc. Persons will be provided with a brief description about the study and receive pre-screening to determine their interest in the study and potential eligibility. Potential participants will be referred to the clinic site for study screening.

Given the long duration of the study, it may be necessary at times to re-assess recruitment strategies. To inform the development and refinement of optimal recruitment methods targeted focus groups including members and opinion leaders of the community likely to be eligible for enrollment in the trial may be convened. These focus groups will be carried out according to the brief protocol described in Appendix 11.

## Screening Evaluation

Screening evaluations will be used to determine the eligibility of each candidate for study enrollment. To be eligible for study enrollment, participants must meet the study eligibility criteria including testing negative for HIV-1 antibodies by commercially available, FDA approved, rapid whole blood HIV-1 EIA. Candidates who fail to meet eligibility criteria by screening evaluations may be re-screened within 45 days of the initial screen if there is a reasonable expectation that the candidate will be eligible after repeat screening. The interval between the screening evaluation and enrollment must not exceed 45 days.

The following procedures and evaluations are to be completed at the screening visit.

Educational Component and Informed Consent

Each potential study candidate presenting for a screening evaluation will first receive a staff-administered educational flip-chart briefing them about the study (Appendix 4) and be given the opportunity to ask questions. Persons interested in participating in the study must then sign a standardized informed consent form (Appendix 5) indicating that they understand what is being asked of them and permitting the collection of epidemiologic data and specimens for clinical laboratory testing before receiving any further screening procedures.

Comprehension Test

Following the educational component and informed consent, potential participants will then be given a brief comprehension test (Appendix 6). Participants will be required to correctly answer all questions before they can be considered eligible for the trial. Potential participants will be given two opportunities to pass the comprehension test before being excluded from enrollment. The test will be administered, and subsequently staff will review the answers and messages with the potential participant. If, in the staff member’s judgment, the participant has a reasonably good grasp of key study concepts and incorrect answers reflect fairly minor misunderstandings, the test can be re-administered the same day. If in the staff member’s judgment, the participant does not appear to grasp key concepts and incorrect answers reflect serious misunderstandings, the participant will receive additional study education and be asked to return on another day for the comprehension test to be re-administered. Once participants completed the informed consent procedures and comprehension test they will be administered the following assessments to establish their study eligibility.

Epidemiologic/Behavioral Assessment

- Screening Form

Clinical Assessment

- Baseline Medical History Form
- Symptom-directed Physical Exam

*Laboratory Assessment*

- Hematology profile (complete blood count [CBC] with differential and platelet count)
- Chemistry profile: albumin, alkaline phosphatase, alanine transaminase (ALT), aspartate aminotransferase (AST), blood urea nitrogen (BUN), calcium, chloride, creatinine, carbon dioxide, direct bilirubin, gamma-glutamyl transpeptidase, glucose, phosphorus, potassium, sodium, total bilirubin, total cholesterol (non-fasting), total protein, uric acid.
- Serum amylase
- Urinalysis
- HIV-1 whole blood rapid EIA
- Hepatitis B surface antigen (HBsAg).
- Rapid plasma regain (RPR)

*HIV risk reduction counseling and testing*

All potential participants will receive standardized pre- and post-test risk reduction counseling and HIV testing by a FDA-approved commercially available whole blood rapid EIA. Reactive rapid HIV test results must be confirmed with both another FDA approved EIA and Western blot (WB) or immunofluorescent assay (IFA). If confirmatory testing yields negative or indeterminate results, follow-up testing should be performed on a blood specimen collected 4 weeks after the initial reactive rapid test result (53).

## Enrollment Visit

HIV-1 negative persons who meet all of the inclusion criteria and none of the exclusion criteria at screening will be scheduled for an enrollment visit within 45 days of screening. The screening and enrollment visits may be spread over 2-3 visits. Study product must be initiated within 24 hours after the enrollment visit for participants randomized to one of the immediate treatment arms.

The following procedures and evaluations are to be performed at the enrollment visit:

*Epidemiologic/behavioral risk assessment*

Study staff will instruct participants on how to complete a 30 minute audio-computer administered self-interview (ACASI). This interview will collect risk behavior, adherence, and other behavioral data at enrollment and designated intervals throughout the study.

Clinical assessment:

- Enrollment form
- Baseline Medical History Form
- Concomitant Medication Form
- Complete Physical Examination

*Laboratory assessment:*

HIV-1 whole blood rapid EIA.

- Chemistry profile
- Hematology profile
- Urinalysis
- Urine for *Neisseria gonorrhoeae* (GC) and Chlamydia trachomatis (CT) testing
- Blood sample for storage

HIV risk reduction counseling

- Standardized HIV risk-reduction counseling by trained staff including study-specific issues:
  - the nature of the study (safety)
  - the unknown level of protection from the product
  - the possible assignment to placebo
  - the importance of avoiding HIV risk behaviors
  - symptoms that may be associated with acute HIV infection

Upon completion of enrollment assessments the investigator should dispense study products to those participants randomized to an immediate treatment arm based on the number assigned in the allocation sequence.

## Product administration

Each participant will be randomly assigned a unique ID number that corresponds to a pre-labeled, blinded, bottle of study product (TDF or placebo) tablets. At the enrollment visit for the immediate treatment arms and at the 9 month visit for the delayed treatment arms, participants will receive 37 tablets. They must return within 1 month after initial product receipt for a safety visit before receiving further study product. At the 1 month (immediate arm) and 10 month (delayed arm) visits participants will **have any returned pills re-dispensed and will** receive **an additional** 68 tablets. At subsequent quarterly visits, participants will receive 105 tablets.

Participants will be instructed to orally self-administer one tablet daily. The tablet should be swallowed whole. It is important to take the tablets regularly. Each dose should be taken as close as possible to twenty-four hours apart at the same time each day.

The participant will be counseled regarding signs and symptoms of toxicity and the importance of adherence. Two weeks and two months after the enrollment visit for the immediate arms and at 9.5 and 11 months for the delayed arms, participants will receive a phone call from study staff to assess any immediate problems. In addition, participants will be encouraged to call for any adverse experiences possibly related to study product, all serious adverse events, symptoms of acute retroviral infection or concerns regarding product adherence. Participants will be instructed to return unused study medication at each study visit and about the correct use of the MEMS® cap. Participants should not share or dispense study product to any other persons.

Dosage Modification

No modification of dosage will be allowed from this study. If a dose is forgotten, it should be taken as soon as remembered on that same day that it was missed. If a daily dose is missed, the participant should not double the dose the next day.

*Adherence Reminder Sessions*

Face-to-face adherence reminder sessions will take place at the initial product dispensing and each study visit thereafter. This session will include:

- The importance of following study guidelines for adherence to once daily study product
- Instructions about taking study pills including dose timing, storage, and importance of taking pills whole, and what to do in the event of a missed dose.
- Instructions about the purpose, use, and care of the MEMS® cap and bottle
- Notification that there will be a pill count at every study visit
- Reinforcement that study pills may be TDF or placebo
- Importance of calling the clinic if experiencing problems possibly related to study product such as symptoms, lost pills or MEMS® cap.

Subsequent sessions will occur at the follow-up visits. Participants will be asked about any problems they are having taking their study pills or using the MEMS® cap.  There will be brief discussion of reasons for missed doses and simple strategies for enhancing adherence, e.g., linking pill taking to meals or other daily activities.  Participants will have an opportunity to ask questions and key messages from the initial session will be reviewed as needed.

## Dual-energy X-ray Absorptiometry (DEXA) Scans

During the screening process, SFDPH participants will be scheduled for a whole body DEXA scan prior to enrollment. All participants who receive a Z-score of less than or equal to 2.0 standard deviations [SD] below the age appropriate mean on the baseline DEXA scan at the total spine, total hip, or femoral neck will have an evaluation of secondary causes performed as part of the study protocol. This work-up will include a thyroid stimulating hormone, 25-hydroxy vitamin D level, testosterone level, a spot urine calcium/creatinine ratio, and additional testing for other secondary causes suggested by the history and physical. Participants with a spot urine calcium/creatinine ratio of > 0.3 will have a 24-hour urine calcium level performed. In addition, participants over the age of 40 will have a serum parathyroid level checked. Participants with an abnormal work-up or with a Z-score of less than -2.5 will be referred to a primary care provider for further work-up and treatment. All participants with a Z-score of less than -2.5 SD at any of the three sites listed above, or participants who are undergoing treatment for secondary causes of low bone density, will not be eligible for enrollment.

Participants will receive subsequent DEXA scans at 12 and 24 months in the immediate arm, at 9 and 24 months in the delayed arm, and at the time of study discontinuation, if at least 6 months has passed since the previous scan. The 9 month DEXA scan in the delayed arm will be performed before the 9 month visit and before initiating study drug if possible. All other follow-up DEXA scans will be performed +/- 1 month of the target visit if possible. If a subsequent DEXA scan detects a > 5% decrease in bone mineral density from baseline at the either the total hip or total spine sites, a work-up for secondary causes (as described above) will be initiated, study product will be discontinued, and participants will continue to be followed at regularly scheduled visits until completion of study visits.

Participants taken off study drug due to low bone density will continue to be followed for the duration of the study. Participants randomized to the delayed arm will have a DEXA scan performed prior to their 9-month visit if possible, but no later than 14 days after initiation of study drug. All previously enrolled participants whose initial DEXA scan demonstrates a Z-score of less than or equal to -2.0 will be evaluated for secondary causes as described above, and if the work-up is abnormal, will be referred to their primary care provider for treatment. Participants with a baseline Z score of less than -2.5 at the total spine, total hip, or femoral neck will remain off study drug but continue follow-up. Participants whose baseline Z scores at all three locations were greater than or equal to -2.5 will be allowed to restart study drug at the discretion of the investigator.

The radiation exposure associated with DEXA scans is relatively low, and comparable to background radiation. Bone density DEXA scans are associated with exposures of approximately 4 mrem per site, similar to the radiation exposure that would be received in a commercial flight across Canada. Whole body DEXA scans for body fat composition assessment are associated with a dose of approximately 0.04 mrem, which is similar to the background radiation received in the course during the course of one day of normal activities.

## Concomitant Medications

At each study visit information regarding concomitant medications/therapies will be solicited from participants and recorded accurately on study CRFs. As participants will be provided STI testing at the study site, STI results and subsequent treatment must be clearly documented on study CRFs. In addition, concomitant medications administered at the time of a serious adverse event will also be collected. Should participants report treatment with any excluded concomitant medication or experimental agent as specified in Section 5.4, study product will be discontinued while on the disallowed concomitant medication.

### Post-exposure Prophylaxis (PEP)

Antiretrovirals for PEP will not be offered as part of this study, however participants may receive them through outside sources. CDC guidelines recommend a 28-day course of PEP for non-occupational exposures with a known HIV-infected or highly-suspected partner within 72 hours of exposure. If participants select to receive PEP outside of this study, they should inform their PEP provider of study participation and stop study product for the duration of the PEP course. Study staff and PEP providers should follow participant’s clinical course. At the conclusion of PEP, healthy HIV-uninfected participants may resume study product. Participants will be specifically asked about PEP at study visits and data (circumstances, antiretrovirals used, follow-up, and HIV status) will be collected.

## Telephone Calls

Once participants receive study product, either at the enrollment visit (immediate arms) or at their 9 month visit (delayed arms) they will arrange with study staff for a scheduled telephone call at 2 weeks and 2 months following study product initiation to report any potential study-related symptoms or problems. If the symptoms resulted in the participant choosing to discontinue study product or require further clinical assessment, the participant will be requested to return promptly to the study site for evaluation and to return unused study product.

## Follow-up Visits

Participants assigned to the immediate treatment arms will be scheduled for follow-up visits at 1 and 3 months after enrollment and then at months 6, 9, 12, 15, 18, 21, and 24 (Table 1, Appendix 3). Study visits for participants assigned to the immediate treatment arm are to be completed + 7 days based on the enrollment visit date. Participants assigned to the delayed treatment arms will be scheduled for follow-up visits at 1 and 3 months after enrollment and then at months 6, 9, 10, 12, 15, 18, 21, and 24 (Table 2, Appendix 3). Study visits for participants assigned to the delayed treatment arm are to be completed + 7 days based on their enrollment visit until their initial dispensing visit . After the initial dispensing visit (month 9), study visits for participants assigned to the delayed treatment arm will be completed + 7 days based on their month 9 visit date. Regularly scheduled evaluations will be made on all participants whether or not they continue to receive study product.

The following procedures are to be performed at all visits unless otherwise specified:

*Epidemiologic/Behavioral Risk Assessment*

- ACASI (3, 6, 9, 12, 15, 18, 21, and 24 months)

Clinical assessment

- Symptom-directed physical examination
- Administration of Adverse Events/Intercurrent Illness Form
- Concomitant Medication Form

*Laboratory assessment:*

- Hematology profile
- Chemistry profile
- Urinalysis
- Blood sample for storage
- Plasma
- Peripheral Blood Mononuclear Cells (PBMCs) (12 months for immediate treatment arms, 21 months for delayed treatment arms only)
- HIV-1 rapid whole blood EIA (3, 6, 9, 12, 15, 18, 21, and 24 months)
- DEXA scan **(SFDPH only: 12 and 24 months for immediate treatment arms, 9 and 24 months for the delayed treatment arms)**
- STI testing
- RPR (12 and 24 months)
- Urine for GC and CT (6, 12, 18, and 24 months)

Standardized HIV risk reduction counseling as per enrollment visit

During the 24 months of the study, additional HIV testing may be requested at any time by study participants, or may be performed as required by the protocol in the case of an indeterminate result or to help determine the timing of HIV infection more precisely.

After participants complete all scheduled study visits, they will be contacted periodically until final study closeout. The purpose of these interim contacts will be to update contact information and to inform participants of the progress of and timeline for completing the study.

## Evaluation of Suspected Acute HIV Infection

Participants who present with signs and symptoms suggestive of acute HIV infection will be evaluated and the following procedures will be performed:

- Symptom-directed physical examination
- Rapid HIV whole blood antibody test with risk reduction counseling emphasizing the increased potential for transmission of HIV during periods of viremia associated with acute infection.

If HIV rapid test is negative, an HIV-ribonucleic (RNA) polymerase chain reaction (PCR) assay will be performed to detect and quantitate viremia. If RNA PCR is also negative, both HIV RNA PCR and rapid HIV antibody testing may be repeated in 4 weeks or sooner at the investigator’s discretion.

The diagnosis of acute HIV infection will require 2 consecutive viral load quantitations of ≥ 5,000 copies/ml in the setting of a negative HIV EIA antibody tests and negative or indeterminate Western Blot (WB) or immunofluorescent assay (IFA) (54). However, any quantifiable viral load will be repeated for confirmation in addition to serial HIV antibody testing and WB assays.

For all participants presenting with suspected acute HIV infection, study product will be continued unless HIV RNA PCR is quantifiable, HIV antibody is detected, or post-exposure prophylaxis is begun. Otherwise, study product will be held for any quantifiable viral load and permanently discontinued upon confirmation of HIV infection. If HIV infection is detected, the participant will be asked to continue in the study in Part B of the protocol (Section 7).

## Early Study Product Discontinuation Visit

If a participant permanently discontinues study product prior to month 24, he will be asked to return to the study site within 72 hours of stopping study drugs for Early Study Product Discontinuation Visit and to return unused study product. These participants will then be asked to continue attending the scheduled study visits through the month 24 visit.

At the Early Study Product Discontinuation Visit, any evaluations showing abnormal results that there is a reasonable possibility of a causal relationship with the study drug should be repeated weekly (or as often as deemed prudent by the investigator) until the abnormality is resolved, returns to baseline, or is otherwise explained.

The following evaluations are to be completed at the early study drug discontinuation visit:

*Clinical assessment*

- Symptom-directed Physical Examination
- Adverse Events/Intercurrent Illness Form
- Concomitant Medication Form
- Status Change

*Laboratory Assessment*

- Hematology profile
- Chemistry profile
- Urinalysis
- Rapid whole blood HIV-1 EIA testing
- Blood sample for storage

*HIV Risk Reduction Counseling*

## Criteria for Suspension of Study Product

Participants will be discontinued from receiving further study tablets for the following reasons:

- Confirmed HIV-1 infection
- Concurrent illness which, in the investigator’s opinion, may be exacerbated by continued product administration, requires treatment which is contraindicated by the protocol, jeopardizes further compliance or follow-up, and may confound the interpretation of adverse events.
- Unacceptable toxicity, as defined in the toxicity management section of the protocol, or which in the judgment of the investigator, compromises the ability to continue study-specific procedures, or is considered to not be in the participant’s best interest.
- Participant request to discontinue for any reason.
- Any clinical condition or social situation that, in the opinion of the investigator, jeopardizes compliance with study product and may confound the interpretation of clinical adverse events
- Non-compliance with at least two consecutive study visits and a clinical or social situation that in the opinion of the investigator would preclude further compliance with study product
- Non-compliance with at least three consecutive study visits and a clinical or social situation that in the opinion of the investigator and CDC would preclude further compliance with study product

Study investigators must make every attempt to continue follow-up visits for the full 24 months whether or not all study medications have been used. Reasons for premature termination from study medication or follow-up visits will be reported on the specific CRF.

## Early Study Termination Visit

If the participant terminates study participation prior to month 24, he will be asked to return to the study site for an Early Study Termination Visit and to return any unused study product. At this visit, any evaluations showing abnormal results that there is a reasonable possibility of a causal relationship with the study drug, should be repeated weekly (or as often as deemed prudent by the investigator) until the abnormality is resolved, returns to baseline, or is otherwise explained.

The following evaluations are to be completed at the early study termination visit:

*Epidemiologic/Behavioral Risk Assessment*

- ACASI

*Clinical assessment*

- Symptom-directed Physical Examination
- Adverse event/Intercurrent Illness Form
- Concomitant Medication Form
- Status Change

*Laboratory assessment*

- HIV-1 whole blood rapid test
- Hematology profile
- Chemistry profile
- Urinalysis
- STI testing
  - RPR
  - Urine for GC and CT
- DEXA scan (for SFDPH subset if > 6 months since last scan)
- Blood sample for storage

## Criteria for Early Study Termination

Participants may be discontinued from the study for the following reasons:

- Participant request to discontinue for any reason.
- Participant non-compliance with study visits or procedures.
- Any clinical condition or social situation that, in the opinion of the investigator, would make the participant unable to comply with the protocol requirements.

Reasons for premature termination from study product or follow-up visits will be reported on the specific CRF.

## Criteria for Suspension of Study

CDC has the right to terminate this study at any time. Reasons for terminating the study may include the following:

1. The incidence or severity of adverse events in this or other studies indicate a potential health hazard to participants
2. Enrollment and/or compliance is unsatisfactory
3. Data recording is inaccurate or incomplete
4. Recommendation by the safety review team

If the study is discontinued before the planned end due to clinical health concerns, each participant remaining in the study at that point will be seen for a final follow-up visit scheduled one month after the last study product had been dispensed. Adverse events, STI, and HIV infections detected through this follow-up visit will be included in the analysis.

## Unblinding

Within 9 months after study database is locked and analysis completed, study staff will receive participant treatment assignment. Participants will be contacted by telephone to be notified of their treatment assignment and be updated on the results of the study.

# STUDY PROCEDURES FOR PARTICIPANTS DIAGNOSED WITH HIV INFECTION (PART B)

In spite of intensive prevention education and risk reduction counseling, it is possible that some participants will become infected with HIV while participating in the study.

Confirmed HIV infection will be defined as the following:

- HIV RNA PCR ≥ 5000 copies/ml on two separate occasions in the setting of a negative HIV EIA antibody test and negative or indeterminate WB or IFA (acute infection)
- Positive HIV EIA antibody test and confirmatory WB or IFA

Participants who have a reactive HIV rapid test or present with a history of a positive HIV test at any time during the study will have confirmatory testing by both a HIV EIA antibody and WB or IFA and RNA PCR (if indicated). Study product will be held for a reactive HIV antibody test. If HIV infection is confirmed, study product will be permanently discontinued.

Participants who develop confirmed HIV-1 infection after enrollment into the study will be discontinued from study product and complete an early termination visit from the main protocol (Part A). They will be referred for medical and psychosocial care. They will be asked to continue in the study (Part B) and be scheduled for study visits at 1, 3, 6, 9, and 12 months after diagnosis of HIV infection (Table 3 – Appendix 3). The termination visit for Part A may also serve as the baseline visit for Part B, with the addition of HIV-specific labs or a separate visit may be scheduled. Following HIV diagnosis, follow-up telephone calls will be made at weeks 1 and 2 according to site-specific procedures and participant request for the purpose of support, referrals, education, and counseling.

## Baseline Visit

The following procedures are to be performed at the time of HIV diagnosis:

Clinical assessment:

- Complete Physical Examination
- Adverse Event/HIV-related Medical History
- Concomitant Medication Form

*Laboratory assessment:*

- HIV EIA Antibody and WB/IFA testing accompanied by risk reduction counseling
- HIV-related tests
  - CD4+ cell count
  - Genotypic and phenotypic resistance testing
  - Viral load by RNA PCR assay (Roche Amplicor Version 1.5)
- Laboratory tests (to be performed if > 2 weeks since last test performed)
  - STI testing
    - Urine for GC and CT
    - RPR
- Chemistry profile
- Urinalysis
- Hematology profile
- DEXA scan
- Blood sample for storage
- Plasma
- PBMCs

## Follow-up Visits

HIV-infected participants will be scheduled for follow-up visits at 1, 3, 6, 9, and 12 months after their baseline visit to assess for antiretroviral resistance and viral setpoints. Study visits are to be completed + 7 days for the month 1 visit and within + 14 days for the remaining protocol-related visit dates based on the enrollment visit date.

The following procedures are to be performed at each follow-up visit unless otherwise specified:

Clinical assessment:

- Symptom-directed Physical Examination
- Concomitant Medication Form
- Adverse event (for events reported during Part A)/Intercurrent Illnesses (new HIV-specific intercurrent illnesses) Form
- HIV risk reduction counseling

*Laboratory assessment:*

- Urinalysis, Hematology and Chemistry Profiles (only if necessary for continuation of AE monitoring from event reported during Part A)
- Viral load by RNA PCR assay (Roche Amplicor Version 1.5)
- CD4+ cell count
- Genotyping[[2]](#footnote-3)
- Blood samples for storage
- Plasma
- PBMCs

After participants complete all scheduled study visits, they will be contacted periodically until final study closeout. The purpose of these interim contacts will be to update contact information and to inform participants of the progress of and timeline for completing the study.

# DATA SOURCES

## Study eligibility assessment

Participants will undergo brief face-to-face interviews at the screening and enrollment study visits by trained study staff. The screening interviews will obtain information on demographics, behavioral risk, and study eligibility criteria.

## Clinical assessment

A symptom-driven physical examination will be performed at screening and a complete physical exam at enrollment by a trained healthcare provider. Clinical symptoms will be systematically assessed in a structured medical interview at 1, 3, 6, 9, 12, 15, 18, 21, and 24 months for the immediate treatment arms, and at 1, 3, 6, 9, 10, 12, 15, 18, 21, and 24 months for the delayed treatment arms. The severity of clinical symptoms will be scored using the established National Institutes of Allergy and Infectious Diseases (NIAID) Division of AIDS (DAIDS) toxicity scale (Appendix 7). Additional data will be collected regarding concomitant medication use.

Telephone interviews will be performed by trained study staff to identify any potential adverse events or adherence issues at 2 and 8 weeks following enrollment for the immediate treatment arms, and at 38 and 44 weeks for the delayed treatment arms.

## Toxicity monitoring

Blood will be obtained at screening, enrollment and then at each subsequent study visit for toxicity monitoring. Routine toxicity monitoring will consist of the following laboratory tests: hematologic profile, chemistry profile, and urinalysis. DEXA scans will be used for a subset of participants. The severity of laboratory toxicity will be assessed and scored according to established laboratory toxicity scales (Gilead Sciences, Inc. toxicity scale for creatinine and DAIDS toxicity scale for all other laboratory toxicity monitoring [Appendix 7]).

## Testing and treatment for sexually transmitted infections (STI)

Participants will be evaluated for syphilis at screening, and *Neisseria gonorrhoeae* (GC) and Chlamydia trachomatis (CT) at enrollment and then at designated study visits during the trial. Although, it is anticipated that the incidence of STIs will be low, the incidence of STIs can be used in conjunction with self-report as a measure of risk behavior. Participants will be evaluated for syphilis using rapid plasma reagin (RPR) and for GC and CT using a urine nucleic acid amplification test. Participants will be referred for treatment if a STI is diagnosed within the study at a venue of their choosing. Treatment will be verified through participant self-report and documented as a concomitant medication at the next study visit.

## HIV-1 testing

HIV-1 serological testing will be performed using an FDA-approved whole blood EIA rapid test at screening, enrollment, and then every 3 months by trained study staff. Additional, HIV serologic testing will be performed at interim visits on participant request. Non-reactive tests will be reported as non-reactive. Participants found to have reactive antibody tests will receive confirmatory testing with both a second FDA-approved EIA and WB or IFA. Participants who present with symptoms suggestive of acute retroviral syndrome will undergo HIV RNA PCR testing and further testing per the algorithm in Section 6.9.

## Behavioral Assessment

Questionnaire Development Systems (QDS) software will be installed at computers at study sites for ACASI use by participants. The ACASI questionnaire will include items on HIV sexual risk behaviors, drug use, depression (55), perceptions of TDF efficacy, adherence, and motivations for study participation. HIV risk behavior data will be obtained at enrollment and at 3 month intervals for the duration of the study. These data include information about unprotected and protected sex with male and female sex partners, sex with HIV seropositive, seronegative, and partners of unknown HIV serostatus, and alcohol and drug use during the past three months. Adherence data will be collected at 3 month intervals (3, 6, 9, 12, 15, 18, 21, 24 months). These adherence items will include a 1 month visual analog scale for dose adherence and timing, (56) questions about reasons for missed doses, possible attitudinal and practical barriers to adherence, the accuracy/truthfulness of self-report, and possible medication sharing.

## Adherence assessments

To enhance validity of data, multiple methods will be used to assess medication adherence including pill count; an electronic medication event monitoring system (MEMS® cap) (57); and ACASI questionnaire items including a one month visual analogue scale (58), reasons for non-compliance, and use of the MEMS® cap. Participants will return the unused tablets and bottle at each follow-up visit. Unused tablets will be counted and recorded on the appropriate CRF. Electronic data collected in the MEMS® cap will be downloaded into a designated, secure study computer.

## Storage of blood specimens

Blood specimens will be stored at all visits beginning with study enrollment. Blood specimens that will be reposited as part of standard specimen collections will include Acid Citrate Dextrose (ACD) or EDTA anticoagulated plasma depending on study visit (as described in appendix 3 – study schedule). These reposited samples may be used for repeat analysis of safety parameters; analysis of viral nucleic acids, antibodies, and antigens, and analysis of tenofovir levels after the study is unblinded. In addition, peripheral blood mononuclear cells (PBMCs) will be collected at enrollment, 6, 12, 18, and 24 month study visits in the immediate arm and at 9, 15, 21, and 24 month study visits in the delayed arm and at all visits from Part B participants. These samples may be used in the future to evaluate other factors associated with host susceptibility to HIV infection or other factors (e.g., TDF) that may modify the risk of HIV acquisition.

## Cryopreservation of blood specimens

Blood specimens will be cryopreserved at enrollment and every three months during the clinical trial and stored at the study site laboratory. Specimens will also be cryopreserved at the seroconversion and post-seroconversion visits. Blood specimens that will be reposited as part of the standard specimen collection protocol will include ACD or EDTA anticoagulated plasma (as described in appendix 3) and peripheral blood mononuclear cells (PBMCs). PBMCs will be viably cryopreserved at visits specified in appendix 3. These specimens may be used for (1) repeat analysis of safety and efficacy parameters; (2) analysis of viral nucleic acids and antigens, including HIV-1 RNA and DNA, HBV DNA and antigens, and other viruses that may be affected by tenofovir; (3) analysis of plasma and intracellular tenofovir levels after the study is unblinded, (4) analysis of humoral immune responses, and (5) analysis of viral phenotypes that may include replication capacity and tropism. Cryopreserved PBMCs may be used for (1) analysis of host antiviral immune responses using ELISPOT, CFC, multiparameter flow cytometry, and other cell-based assays as required to determine if infection during tenofovir prophylaxis is associated with more robust antiviral immune responses, and (2) host genotyping of loci known to affect antiviral immune responses, plasma RNA level, or rates of progression which may include HLA loci, CCR5, and SDF. All laboratory testing of safety and efficacy endpoints will be performed by laboratories that routinely provide clinical diagnostic testing in compliance with locally applicable regulations (e.g. CLIA if in the U.S Specimens referred to laboratories will be labeled with study codes and will not include any participant name identifiers).

Referral of specimens for repeat analysis of safety or efficacy parameters will be performed at the discretion of the principal investigator, which would be prompted by evidence of failed proficiency testing at the designated laboratory for this trial, or evidence that the selected assay systems may have systematically failed to identify clinically relevant toxicity. The host genotyping, immune studies, and nucleic acid studies would be performed for research use only and results would not be shared with the participants’ physicians. Use of the specimens during the blinded phase of the study will require approval of the principal investigator, who will take steps to assure that the proposed analysis would not compromise the blinding of the study.

Study participants will be informed that the study involves specimen storage for additional laboratory research required to understand the effects of tenofovir chemoprophylaxis on viruses and the body’s responses to infections. Test results from reposited specimens will not be reported to the participant.

**Table 2. Specimens to be C**ryopreserved

| **Specimens** | **Analyses that may be performed** | **Visit** |
| --- | --- | --- |
| Plasma | Repeat safety parameters  Analysis of HIV nucleic acids, antigens and phenotypes. Analysis of other viral nucleic acids, antigens, and phenotypes, possibly to include HBV and HCV.  Plasma tenofovir levels  HIV viral load and resistance testing  Humoral immune responses | Enrollment, month 1, every 3 months thereafter, Seroconversion, every post-seroconversion visit. |
| PBMCs | Analysis of host antiviral immune responses using ELISPOT, Cytokine flow cytometry, multiparameter flow cytometry and other cell based assays as required to determine if viral exposure during tenofovir prophylaxis is associated with more robust antiviral immune responses  Host genotyping of loci known to affect antiviral immune responses, plasma RNA level, or rates of disease progression  Intracellular concentrations of active metabolites of tenofovir | Immediate arm visits: enrollment, 6, 12, 18, and 24 month visits  Delayed arm visits: 9, 15, 21, and 24 month visits, Seroconversion, every post-seroconversion visit |

# ADVERSE EVENTS

## Adverse Events

An adverse event (AE) is any untoward medical occurrence in a patient or clinical investigation participant administered a pharmaceutical product, regardless of causality assessment. An AE can therefore be any unfavorable and unintended sign, symptom, or disease temporally associated with the use of a medicinal product, whether or not considered related to the medicinal product. Pre-existing events, which increase in frequency or severity or change in nature during or as a consequence of use of a drug in human clinical trials, will also be considered as adverse events. AEs may also include pre‑ or post‑treatment complications that occur as a result of protocol-mandated procedures (e.g., invasive procedures such as biopsies).

An AE does not include:

- Medical or surgical procedures (e.g., surgery, endoscopy, tooth extraction, transfusion); the condition that leads to the procedure is an adverse event.
- Preexisting diseases or conditions present or detected prior to start of study product administration that do not worsen.
- Situations where an untoward medical occurrence has not occurred (e.g., hospitalization for elective surgery, social and/or convenience admissions).
- Overdose of either study drug or concomitant medication without any signs or symptoms unless the participant is hospitalized for observation.

Any medical condition or clinically significant laboratory abnormality with an onset date before the first **screening visit** is considered to be preexisting, and should be documented in the appropriate CRF. Any new event, exacerbation of a preexisting condition, or new chronic condition with an onset date after the initial screening visit but before the enrollment visit date should be documented as an intercurrent illness or new chronic condition on the appropriate CRF.

Any AE (i.e., a new event or an exacerbation of a preexisting condition) with an onset date after the enrollment visit date up to the last day on study (including any time off study medication and follow‑up), should be recorded as an AE on the appropriate CRF. If any AE occurs on study, the participant will be referred for appropriate treatment and medical supervision. All AEs judged to be clinically significant and possibly or probably related to study product, including clinically significant laboratory abnormalities, will be followed until they are no longer clinically significant or have reached a steady state.

## Assessment of Adverse Events

Study participants will be instructed to contact the clinic in the event of any AE possibly related to study product except for life-threatening AEs, for which participants will be instructed to seek immediate emergency care. Depending on the severity and seriousness of the event, the study staff will instruct the participant to visit the study site office (for more mild events) or go to an emergency room (for more serious events) for immediate evaluation. In the event of a life-threatening AE, the participant should notify study staff as soon as it is medically feasible.

All AEs occurring during the study will be assessed by the investigator and recorded on the appropriate CRF. The study site clinician/investigator must provide on this form information on symptoms, time/date of onset, severity, frequency, relationship to study product, action(s) taken, and participant outcome. With appropriate permission of the participant, records from off-site medical visits related to all possible and probable study-related AEs and SAEs will be obtained, and required data elements will be recorded on this AE CRF. All participants reporting AEs will be followed clinically, until the AE resolves or stabilizes as per the appropriate toxicity algorithm.

## Serious Adverse Events

A serious adverse event (SAE) is any untoward medical occurrence that at any dose (including overdose):

- Is fatal
- Is life-threatening
- Requires or prolongs inpatient hospitalization
- Results in permanent or significant disability/incapacity
- Is a congenital anomaly/birth defect of a participant who received study drug
- Other: Important medical events that may not result in death, be immediately life-threatening, or require hospitalization, may be considered a SAE when, based upon appropriate medical judgment, they may jeopardize the participant and may require medical or surgical intervention to prevent one of the outcomes listed in this definition. Examples of such events are:
- Intensive treatment in an emergency room or at home for allergic bronchospasm
- Blood dyscrasias or convulsions that do not result in hospitalization

Clarification of Serious Adverse Events

- Death is an outcome of an adverse event, and not an adverse event in itself.
- All deaths, regardless of cause, must be reported for participants on study and for deaths occurring within 30 days of last study drug dose or within 30 days of last study evaluation, whichever is longer.
- “Occurring at any dose” does not imply that the participant is receiving study drug at the time of the event. Product use may have been interrupted temporarily prior to the onset of the SAE, but may have contributed to the event.
- “Life‑threatening” means that the participant is at immediate risk of death from the event as it occurred. This does not include an event that might have led to death, if it had occurred with greater severity.
- Complications that occur during hospitalizations are AEs. If a complication prolongs hospitalization, it is a SAE.
- “Inpatient hospitalization” means the participant has been formally admitted to a hospital for medical reasons, for any length of time. This may or may not be overnight. It does not include presentation and care within an emergency department unless the participant is “admitted”.
- The investigator should attempt to establish a diagnosis of the event based on signs, symptoms and/or other clinical information. In such cases, the diagnosis should be documented as the AE and/or SAE and not the individual signs/symptoms.

## Serious Adverse Event Reporting Requirements

All Serious Adverse Events

The CRO and CDC must be notified within 24 hours regarding the occurrence of any SAE that occurs after the start of study. SAEs may first be reported via telephone or fax. The procedures for reporting all SAEs, regardless of causal relationship, are as follows:

Record the SAE on the AE CRF and complete the “Serious Adverse Event Report” form.

- Fax the serious adverse event report to both the CRO and CDC within 24 hours of the investigator’s knowledge of the event.
- For fatal or life‑threatening events, also fax copies of hospital case reports, autopsy reports, and other documents when requested and applicable.

| CRO Medical Monitor: | Joan Drucker, MD  Phone: (919) 403-1019  (919) 313-1717/ (919) 313-1706 Fax: Constella Group, Inc.  Attn: Sherry Jones/Calvin Motley  (919) 869-1654 E-mail: [jdrucker@constellagroup.com](mailto:jdrucker@constellagroup.com)  [sjones@constellagroup.com](mailto:sjones@constellagroup.com) |
| --- | --- |
| CDC Medical Officer: | Lisa Grohskopf, MD, MPH  Phone: (404) 639-611~~7~~6/ (404) 639-1948 Fax: (404) 639-6127  Lisa Grohskopf/Brandi Collins E-mail: lgrohskopf@cdc.gov |

CDC may request additional information from the investigator to ensure the timely completion of accurate safety reports.

The investigator should identify all therapeutic measures necessary for resolution of the SAE. Any medications necessary for treatment of the SAE must be recorded onto the concomitant medication section of the participant’s CRF.

Preliminary reports of deaths, life-threatening, or serious and unexpected AEs will be followed by detailed descriptions on the SAE CRF and will include copies of hospitalization summaries, pathology reports, operative reports, laboratory reports, autopsy reports and/or other documents when applicable. Follow‑up of ongoing SAEs and clinically significant AEs that are possibly or probably related to study product will continue up to 30 days after study product discontinuation or until the site investigator and/or CDC’s medical officer determine that the participant’s condition is stable, whichever is longer. CDC may request that certain adverse events be followed until resolution.

Post-Study Reporting Requirements

All deaths, regardless of cause or relationship, must be reported for participants on study and for deaths occurring within 30 days of last study product dose or within 30 days of last study evaluation, whichever is longer.

Investigator Reporting Requirements for SAEs

A SAE may qualify for reporting to regulatory authorities if the SAE is possibly attributable to the study drug, and is unexpected/unlisted based upon the current Investigator’s Brochure. In this case, all investigators will receive a formal notification describing the SAE.

The site principal investigator should notify their local Institutional Review Board (IRB) in accordance with the local institutional policy.

## Clinical Laboratory Abnormalities and other Abnormal Assessments as Adverse Events or Serious Adverse Events

Laboratory abnormalities will be captured and graded in the laboratory database. Laboratory abnormalities (e.g., clinical chemistry, hematology, or urinalysis) that are grade 3 or above on the DAIDS toxicity scale, or require a concomitant medication**,** lead to study drug interruption or discontinuation, are associated with symptoms, or if the investigator otherwise feels the abnormality is clinically significant must be recorded as an AE, as well as a SAE if applicable. If the laboratory abnormality is part of a syndrome, record the syndrome or diagnosis. Other abnormal assessments (e.g., electrocardiogram, X‑rays, vital signs) that are associated with signs and/or symptoms must be recorded as an AE and SAE if they meet the criteria as described in Sections 9.1 and 9.3.

## Toxicity Management

All adverse events and abnormal laboratory values (excluding creatinine elevations and decreases in BMD) will be graded according to the NIAID DAIDS Toxicity Grading Scale (Table 1 - Appendix 7). Creatinine elevations will be managed using the Gilead NIAID modified toxicity scale (Table 2 – Appendix 7). Decreases in BMD for the subset of participants receiving DEXA scans will be managed as described in section 6.5. All other clinical and laboratory toxicities will be managed according to the toxicity algorithms detailed in Appendix 8. Any questions regarding toxicity management should be directed to the CRO Medical Monitor and the CDC Medical Officer.

Grade 1 and 2 Laboratory Abnormality or Clinical Event (excluding creatinine elevations)

Continue study product at the discretion of the site investigator.

Grade 3 Laboratory Abnormality or Clinical Event (excluding creatinine elevations)

- Unrelated to study product

For a grade 3 laboratory abnormality or clinical event, confirmed by repeat testing, study product may be continued at the discretion of the site investigator if the event is considered to be unrelated to study product.

- Possibly related to study product

For a grade 3 clinical event or laboratory abnormality, confirmed by repeat testing, of which toxicity is possibly related to study product, study product should be withheld until the toxicity returns to ≤ grade 1, at which point the participant can be re-challenged with study drug at the site investigator’s discretion.

If a toxicity recurs to ≥  grade 3 following re-challenge with study product, is confirmed, then study product should be permanently discontinued and participants monitored and managed according to local practice.

- Probably related to study product

For a grade 3 clinical event or laboratory abnormality, confirmed by repeat testing, of which toxicity is probably related to study product, study product should be discontinued and clinical management should be discussed with the CDC medical project officer or the CRO Medical Monitor.

If a grade 3 laboratory toxicity is not confirmed by repeat testing, it should be managed per algorithm for the new toxicity grade.

*Grade 4 Laboratory Abnormality or Clinical Event (excluding creatinine elevations)*

For a grade 4 clinical event or laboratory abnormality confirmed by repeat testing, study product should be discontinued and participant monitored and managed according to local practice. The participant should be followed as clinically indicated until the event resolves to baseline, stabilizes, or is otherwise explained, whichever occurs first.

If a grade 4 toxicity is confirmed and is considered to be unrelated to study product, study product should be withheld until the event resolves to baseline. Study product can then be restarted at the discretion of the site investigator.

If a grade 4 laboratory toxicity is not confirmed by repeat testing, it should be managed per algorithm for the new toxicity grade.

*Creatinine Elevations*

For a grade 1 serum creatinine elevation (>.5 increase from enrollment level) confirmed by repeat testing, study product should be held until creatinine returns to within .3 mg/dL of enrollment level, at which point participant can be re-challenged with full dose of study drug. If a confirmed grade 1 toxicity reoccurs, study product should be permanently discontinued, and participant monitored until level returns to baseline or stabilizes.

For a grade > 2 serum creatinine elevation (serum creatinine > 2.1) confirmed by repeat testing, study product should be permanently discontinued, and participant monitored until level returns to baseline or stabilizes.

Relationship of adverse event to study product

The Investigator must determine the relationship of the AE to the study product. For each AE, an assessment of the relatedness to the test agent should be made using the following scale:

- Unrelated: Onset of the AE had no reasonable temporal relationship to administration of the study product or a causal relationship to administration of the study product is biologically implausible or the event is attributed to an alternative etiology.
- Possibly Related: Onset of the AE has a reasonable temporal relationship to study product administration and a causal relationship is not biologically implausible.
- Probably Related: Onset of the AE has a strong temporal relationship to administration of the study product that cannot be explained by the participant’s clinical state or other factors and a causal relationship is not biologically implausible.
- Definitely Related: Onset of the AE shows a distinct temporal relationship to administration of the study product that cannot be explained by the participant’s clinical state or other factors or the AE occurs on rechallenge or the AE is a known reaction to the product or chemical group or can be predicted by the product’s pharmacology.

These criteria in addition to good clinical judgment should be used as a guide for determining the causal assessment. If it is felt that the AE is not related to study product, then an alternative explanation should be provided.

# STATISTICAL CONSIDERATIONS

## Study Design and Analysis Plan Overview

This is a phase II randomized double-blinded placebo-controlled trial to assess the clinical and behavioral safety of daily oral use of TDF among MSM. Four hundred MSM at risk of HIV-1 infection will be recruited from two sites, randomized 1:1:1:1 to one of four treatment arms, and followed for 24 months. Two treatment arms will receive study product (TDF or placebo) for the entire 24 months (the immediate treatment arms) and the other two treatment arms will receive no study product for the initial 9 months and then receive either TDF or placebo for the remaining 15 months. The data analysis will compare clinical and behavioral safety data between the study arms. The analysis will employ the intent-to-treat principle whereby all data from enrolled participants will be analyzed according to the initial randomization assignment regardless of how many doses they received.

## Endpoints

### Primary Endpoints

- The clinical safety and toxicity of TDF will be assessed by the occurrence of moderate and severe clinical and laboratory adverse events in each treatment group. Mild event rates will also be reported.
- The behavioral safety will be assessed by evaluating the change in the reported type and frequency of sexual risk behaviors (e.g. unprotected anal sex, number of sex partners) in each treatment group.

### Secondary Endpoints

Secondary endpoints will include:

- assessment of adherence
- incidence of HIV infection
- incidence of antiretroviral drug resistance among those individuals who become HIV-infected over the course of the study

## Sample Size and Power Calculations

Recruitment will target 400 healthy, HIV-uninfected MSM that will be equally distributed across four treatment arms. Based on previous HIV prevention studies of similar length among MSM, it is estimated there will be an approximately 10-20% loss to follow-up over the study period.

Within the constraint of four study arms (TDF vs. placebo by immediate vs. delayed) and a total

study size of 400 participants, two design issues were explored: allocation of participants to the

four study arms and amount of time needed before the delayed arms began receipt of study

product. These evaluations were focused on the need to achieve a balance between good statistical properties (i.e., power) for the evaluation of both clinical AEs and behavioral outcomes as well as satisfy other criteria such as maximizing participation, especially in the delayed arms.

Equal allocation of participants to each study arm was found to be the best overall allocation scheme given the goals of the study. Additionally, a nine-month lead-in period for the delayed arm provided the best compromise between concerns for statistical power for comparisons of behavioral outcomes and logistical problems related to recruitment.

Sample size calculations for safety are expressed in the terms of the ability to detect SAEs. The following tables show the probabilities of observing, 0, 1, 2, 5+,  8+ or 10 or more SAEs among groups of n=100 (the immediate arm) and n=200 (both arms) for a range of possible true event rates. For example, if the true SAE rate is 3%, the probability of observing five or more events is 18.2% with n=100 and 71.9% with n=200.

**Event Probabilities for n=100**

| Event Rate  (SAE) | Probability of | | | | | |
| --- | --- | --- | --- | --- | --- | --- |
|  | 0 events | 1 event | 2 events | 5+ events | 8+ events | 10+ events |
| 1% | 0.366 | 0.370 | 0.185 | 0.003 | 0 | 0 |
| 2% | 0.133 | 0.271 | 0.273 | 0.051 | 0 | 0 |
| 3% | 0.048 | 0.147 | 0.225 | 0.182 | 0.010 | 0 |
| 4% | 0.017 | 0.070 | 0.145 | 0.371 | 0.048 | 0.007 |
| 5% | 0.006 | 0.031 | 0.081 | 0.564 | 0.128 | 0.128 |
| 10% | 0 | 0 | 0.002 | 0.976 | 0.794 | 0.549 |
| 15% | 0 | 0 | 0 | 1.000 | 0.999 | 0.988 |

**Event Probabilities for n=200**

| Event Rate  (SAE) | Probability of | | | | | |
| --- | --- | --- | --- | --- | --- | --- |
|  | 0 events | 1 event | 2 events | 5+ events | 8+ events | 10+ events |
| 1% | 0.134 | 0.271 | 0.272 | 0.052 | 0.001 | 0 |
| 2% | 0.018 | 0.072 | 0.146 | 0.371 | 0.049 | 0.049 |
| 3% | 0.002 | 0.014 | 0.043 | 0.719 | 0.254 | 0.081 |
| 4% | 0 | 0.002 | 0.010 | 0.905 | 0.550 | 0.281 |
| 5% | 0 | 0 | 0.002 | 0.974 | 0.787 | 0.545 |
| 10% | 0 | 0 | 0 | 1.000 | 0.999 | 0.996 |
| 15% | 0 | 0 | 0 | 1.000 | 1.000 | 1.000 |

The Blyth-Still-Casella 95% confidence intervals based on observing 0, 1, 2, 5, 10 events in each group are given in the following table.

**Two-sided 95% Confidence Intervals for Various SAE Rates**

| Sample Size | 0 events | 1 event | 2 events | 5 events | 10 events |
| --- | --- | --- | --- | --- | --- |
| N=100 | (0, 3.5%) | (0, 4.9%) | (0.4%, 6.6%) | (2%, 11%) | (4.9%, 17.1%) |
| N=200 | (0, 1.7%) | (0, 2.5%) | (0.2%, 3.5%) | (1%, 5.4%) | (2.6%, 8.7%) |

Power is calculated based on the exact test (or likelihood ratio test).  The type 1 error is assumed to be 0.05.  The following table shows the power to detect the difference with various SAE rates in the treatment group given SAE rate in placebo group equals 0.01.

**Power Calculation**

| SAE Rates in Treatment Group  (n=200) | Power |
| --- | --- |
| 0.03 | 17% |
| 0.04 | 36% |
| 0.05 | 56% |
| 0.06 | 73% |
| 0.07 | 85% |

## Methods of Analysis

Analysis Populations

Intent‑to‑Treat (ITT) Population

This population will include all participants who are randomized into the study and receive at least one dose of study medication. The exclusion of participants who do not receive study drug is justified by the fact that the decision to not initiate study medication is made with no knowledge of the randomized treatment. Participants with major eligibility violations that are identifiable based on pre-randomization characteristics will be excluded. Participants who receive study medication other than that intended will be analyzed according to the group to which they were randomized.

Analysis of the Conduct of the Study

This population will include all screened participants. Specifically, summaries of the following endpoints will be generated: participant screening, eligibility, participant enrollment, participant disposition and reasons for discontinuation, participant medication compliance, participant compliance with study visits, and protocol violations/deviations. Simple descriptive statistics, including percentages, means, and quartiles will be used to summarize the distribution of these endpoints. In addition, the above analyses will be carried out by clinical site to identify any potential site-specific issues. Participants’ disposition will be tabulated as screened but never enrolled, enrolled but never initiated study product, on-study and continuing with study product, discontinued from study product but continuing follow-up for HIV infection or off-study and not continuing follow-up.

*Types of Outcomes and Statistical Analyses*

The outcomes of interest and their respective analyses can be classified by the following factors:

Type of outcome and number of responses per individual:

A) Time until first occurrence of some specified event –

One response per individual – Cox survival regression

B) Yes/no outcome;

One response – Logistic regression

Multiple responses – Longitudinal binary regression

C) Continuous outcome

In general, counts (e.g. number of partners) will be treated as Poisson distributed

outcomes while laboratory values or transformations of those values (e.g. creatinine level,

log (creatinine level) will be treated as normally distributed outcomes.

One response – Poisson, linear regression

Multiple responses – Longitudinal Poisson, linear regression

All statistical analyses will be conducted using SAS© version 8.2 using PROC PHREG (survival analyses) and PROC GENMOD (logistic, Poisson, linear regressions for single or multiple responses). The analyses with multiple responses per individual will be treated as longitudinal regression models that will take into account that the responses tend to be correlated within individuals.

## Safety Analyses for Clinical and Laboratory Measurements

The rate of severe reactions will be used as the primary measure of clinical safety of this study drug. Assessment of product safety will include clinical observation and monitoring of hematologic and chemical parameters. All participants will be monitored for local and systemic adverse reactions during the course of the trial. Participants will be closely monitored for the first 8 weeks after product dispensation and followed for at least 15 months on product.

Adverse experiences will be coded into MedDRA (Medical Dictionary for Regulatory Activities) preferred terms. The number and percentage of participants experiencing each specific adverse experience will be tabulated by severity and relationship to treatment. Overall summaries by treatment will include the number and percentage of participants experiencing: (1) an adverse experience; (2) any moderate, severe, or life-threatening experience; (3) any severe or life-threatening experience; (4) adverse experience judged possibly related, probably related, or definitely related to treatment. Additional summaries will summarize the effect of adverse events on study drug dosing. A separate tabulation of SAEs will also be generated and will provide details including severity, relationship to treatment, onset, duration, and outcome. Adverse events that lead to product or study discontinuation will be listed separately.

Participants who become HIV-1 infected while on study will be monitored in a second part of the protocol (Part B). Both potential benefits and potential detrimental effects of the study product based on secondary endpoint analyses described above will be investigated. Clinical endpoints (major and minor) associated with the disease progression (e.g., setpoint, resistance,) will be recorded. Because few clinical endpoints are expected to be observed to support a quantitative analysis, qualitative case-descriptions will be done.

Three types of safety analyses will be performed depending on the outcome of interest as described in the next three sections. All safety analyses described will be adjusted for study site and the delayed /immediate treatment group variable. It will be explored whether these factors modify (i.e., effect modifiers) any of the observed outcome – treatment relationships and whether study drop-outs are comparable to either a) all others still on study (missing completely at random) or b) to others still on study within their site by study group (missing at random).

## Safety Analyses for Behavioral Outcomes

Data will be collected on HIV risk behaviors at enrollment and 3-month intervals throughout the study to assess the behavioral safety of study product use. The main behavioral safety analysis will compare the overall level of selected behaviors in the immediate and delayed treatment arms over the first nine months of the study. Statistical analyses for dichotomous (yes/no) responses will account for the within-individual correlation using longitudinal binary regression models. The study will have good power (>80%) to detect differences in the probability of a yes response between the groups of 12% or more assuming that the probability of a yes response in the delayed group is 28% or greater. All analyses will account for within-individual correlation using longitudinal models for continuous variables (e.g. Poisson outcomes). Subsequent analyses will examine whether there is a trend in the difference of a yes response between the two groups over time and trends in the probability of a yes-response within both the TDF and placebo groups while on study product. This last analysis will also be examined in relation to an individual’s perception that they have been randomized to a TDF group.

In all analyses, study drop-outs will be evaluated to assess their comparability to either a) all others still on study (missing completely at random) or b) to others still on study within their site by study group (missing at random).

## Analyses for Study Product Acceptability

The following data will be collected at 3-month intervals regarding acceptability and compliance of usage of the study product:

1) Manual pill count;

2) Electronic cap monitoring (MEMS cap)

Number of times bottle has been opened;

Date/time of each opening;

3) Self -reported adherence behavior and perceptions of study product (ACASI)

Study pill compliance and acceptability will be analyzed using several key measures including the percentage of expected doses actually taken in a given time period; consistency of study product usage over time; and by examining relationships between compliance and key participant demographic, clinical, and psychosocial characteristics.

The major measurement of interest for each three-month period will be the percentage of study product utilized as measured by the MEMS cap device, but pill count and self-reported data on adherence will also be used to address inconsistencies.

The major comparisons of interest for compliance are the differences in usage for the TDF and placebo groups while on study product. Comparisons involving the first 15 months on drug will use all TDF vs. placebo participants while comparisons after 15 months on product will be limited to the participants in the immediate arm. The statistical analyses will utilize longitudinal continuous regression models that can account for within-individual correlation.

Power calculations indicate that while a one-time period with 100 participants per group requires a fairly large difference in compliance (10% vs. 25% noncompliance, power=81%) for power >80%. However, comparisons based on more time periods or 200 participants per group fair considerably better. For example, with 5 time periods and 100 per group (common correlation among time periods assumed to be 0.5) approximate 80% power would be achieved under a true difference of 8% vs. 18%. For 200 participants in each group and 3 time periods, 8% vs. 16% non-compliance in the two groups would have power equal to 86%.

## Independent Safety Review Team

A safety review team will review the progress and clinical and behavioral safety profile of this study while the study is ongoing. Safety data collected at each site will be reviewed rapidly by the investigators and CDC. The safety review team will convene at scheduled timepoints 6, 12, and 18 months following study initiation to examine the safety results of the trial and also focus on logistical issues such as accrual, retention, quality of clinical and laboratory data, and implications of results of external studies.

All serious adverse events (SAE) that are study related and unexpected will be provided to the safety review team at their regular safety reviews, and should any reportable SAEs occur, these will be reported to the safety review team at the same time as they are reported to the IRBs.

As TDF is a FDA-approved licensed drug that has already been evaluated in numerous previous safety studies, no formal stopping rules will be provided to the safety review team. However, ad-hoc meetings of the safety review team will be triggered by a) > two grade 4 adverse events of the same nature (e.g., two cases of hepatic failure) that are definitely or probably related to study product; b) > three grade 3 adverse events of the same nature that are definitely or probably related to study product. When reviewing the data at regularly scheduled time points or at ad-hoc reviews, the safety team will then be asked to make a clinical assessment to determine if the nature, frequency, and severity of adverse effects associated with a study regimen warrant the early termination of the study in the best interests of the participants.

## Analysis Schedule

The primary safety analysis will be conducted within 6 months after the last HIV uninfected participant completes 24 months on study.

# RESPONSIBILITIES

## Investigator responsibilities

Compliance with laws and regulations

This study will be conducted in accordance with U.S. Food and Drug Administration (FDA), the international council of harmonization (ICH), the Declaration of Helsinki, and local ethical and legal requirements. The investigator will ensure that the basic principles of “Good Clinical Practice,” as outlined in 21 CFR 312, subpart D, “Responsibilities of Sponsors and Investigators,” 21 CFR, part 50, and 21 CFR, part 56, are adhered to.

Institutional Review Board (IRB) Approval

This protocol and any accompanying material to be provided to the participant (such as advertisements, participant information sheets, or descriptions of the study used to obtain informed consent) will be submitted to both the local and CDC IRBs. Both IRB approvals must be obtained before starting the study and should be documented in a letter to the investigator specifying the protocol number, protocol version, documents reviewed, and date on which the committee met and granted the approval. Any modifications made to the protocol after receipt of IRBs’ approval must also be submitted to the IRBs for approval prior to implementation.

Informed Consent

It is the responsibility of the investigator or designee to obtain written informed consent from each individual participating in this study after adequate explanation of the aims, methods, objectives, and potential hazards of the study and prior to undertaking any study-related procedures. The investigator or designee must utilize the IRB‑approved consent forms for documenting the written informed consent. Each informed consent will be appropriately signed and dated by the participant and the person obtaining consent.

Confidentiality

The investigator must assure that participants’ anonymity will be strictly maintained and that their identities are protected from unauthorized parties. Only participant initials and an identification code (i.e., not names) should be recorded on any form submitted to CDC, the CRO, and IRBs.

The investigator agrees that all information received from Gilead, including but not limited to the Investigator Brochure, study product and any other study information remain the sole and exclusive property of Gilead during the conduct of the study and thereafter. This information is not to be disclosed to any third party (except employees or agents directly involved in the conduct of the study or as required by law) without prior written consent from Gilead. The investigator further agrees to take all reasonable precautions to prevent the disclosure by any employee or agent of the study site to any third party or otherwise into the public domain.

Study Files and Retention of Records

The investigator must maintain adequate and accurate records to enable the conduct of the study to be fully documented and the study data to be subsequently verified. These documents should be classified into 2 separate categories: (1) investigator’s study file, and (2) participant clinical source documents.

The investigator’s study file will contain the protocol/amendments, CRF and query forms, IRB and governmental approval with correspondence, informed consent, drug records, staff curriculum vitae and authorization forms, safety notification letters, and other appropriate documents and correspondence.

Participant clinical source documents (usually defined by the project in advance to record key efficacy/safety parameters independent of the CRFs) would include the following: participant hospital/clinic records; physician’s and nurse’s notes; appointment book; original laboratory reports; electrocardiogram (ECG); electroencephalogram (EEG); X‑ray, pathology and special assessment reports; consultant letters; screening and enrollment log; etc.

All clinical study documents must be retained by the investigator until at least 2 years after the last approval of a marketing application and until there are no pending or contemplated marketing applications. However, if no application is filed or if the application is not approved for such indication, all clinical study documents must be retained until 2 years after the investigation is discontinued and regulatory authorities have been notified. Investigators may be required to retain documents longer if required by applicable regulatory requirements or an agreement with CDC and/or Gilead. The investigator must notify CDC prior to destroying any clinical study records.

Should the investigator wish to assign the study records to another party or move them to another location, CDC must be notified in advance and give approval.

If the investigator cannot guarantee this archiving requirement at the study site for any or all of the documents, special arrangements must be made between the investigator and CDC to store these documents in sealed containers outside of the site in order that they can be returned sealed to the investigator in case of a regulatory audit. Where source documents are required for the continued care of the participant, appropriate copies should be made for storage outside of the site.

Case Report Forms (CRFs) and Source Documentation

For each participant screened, a CRF must be completed and signed by the study staff member administering the form. If a participant withdraws from the study, the reason must be noted on the CRF. If a participant is withdrawn from the study because of a treatment-limiting adverse event, thorough efforts should be made to clearly document the outcome.

Study Product Accountability

The study sites will receive a supply of study products (TDF and placebo) sufficient for the anticipated number of study participants. All supplies must be stored in a limited access area that is securely locked. For purposes of inventory accountability, the study sites will not make supplies of the study product available for distribution by any person not part of the study staff or provide these supplies to persons not enrolled in the study.

Each investigator or designee is responsible for ensuring adequate accountability of all used and unused study product. This includes acknowledgment of receipt of each shipment of study product (quantity and condition) and participant dispensing records. Dispensing records will document quantities received from Gilead and/or CDC and quantities dispensed to participants, including lot number, date dispensed, participant identifier number, participant initials, and the initials of the person dispensing the medication. All unused supplies of the study product must be furnished to CDC at the end of the study, or disposed of in a manner specified by CDC. Prior to return of used, unused and depleted containers, final drug accountability and reconciliation will be performed by the monitor.

All study drug supplies and associated documentation will be regularly reviewed and verified by the monitor.

Inspections

Accurate, consistent, and reliable data will be insured through the use of standard GCPs. The study will be monitored for compliance with US FDA regulations and GCP guidelines by local monitors and CRO. Investigators at CDC and at each trial site will permit trial-related monitoring, audits, IRB review, and regulatory inspections by providing direct access to source data and study documents.

Protocol Compliance

The investigators are responsible for ensuring the study is conducted in accordance with the procedures and evaluations described in this protocol.

## Sponsor Responsibilities

Protocol Modifications

All protocol modifications must be approved by CDC and submitted to the CDC IRB and local IRBs. Both CDC and local IRB approvals must be obtained before protocol changes may be implemented.

*Clinical Monitoring*

Site visits will be conducted by CDC staff and by the clinical monitors of a designated CRO. The purpose of the clinical monitoring is to assure the quality and accuracy of data collected on the CRFs and entered in the study database, and to determine that all regulatory requirements surrounding the clinical trial are met.

Before the study begins, the CRO clinical monitors will conduct an initiation visit at each site. Following this visit, regular monitoring visits will be established, and the end of the study will be followed by a close-out visit. The overall responsibility of the monitors is to assist the CDC in ensuring that the study is being conducted according to the protocol, ICH/GCP and applicable regulatory requirements.

A detailed monitoring plan will be developed for this study and will be used by all clinical monitors. The plan will specify the responsibilities and qualifications of the identified clinical monitors, back-up provisions, in-house monitoring procedures, and site monitoring procedures. All monitoring visits will be documented.

Study Report and Publications

Presentation and publication of the results of this study will be governed by guidelines and policies determined by the CDC investigators and their collaborators at ARCA, SFDPH, and FCH. Any presentation, abstract, or manuscript will be submitted to CDC for review and approval prior to submission.

## Joint Investigator/Sponsor Responsibilities

Access to Information for Monitoring

In accordance with International Conference on Harmonization Good Clinical Practice (ICH‑GCP) guidelines, the study monitor must have direct access to the investigator’s source documentation in order to verify the data recorded in the CRFs for consistency.

The monitor is responsible for routine review of the CRFs at regular intervals throughout the study, to verify adherence to the protocol, and the completeness, consistency and accuracy of the data being entered on them. The monitor should have access to any participant records needed to verify the entries on the CRFs. The investigator agrees to cooperate with the monitor to ensure that any problems detected in the course of these monitoring visits are resolved.

Study Discontinuation

Both CDC and the investigator reserve the right to terminate the study at any time. Should this be necessary, both parties will arrange discontinuation procedures. In terminating the study, CDC and the investigator will assure that adequate consideration is given to the protection of the participants’ interests.

Study Completion

The following data and materials are required by CDC before a study can be considered complete or terminated:

- Laboratory findings, clinical data, and all special test results from screening through the end of the study follow-up period
- Complete and corrected CRFs properly completed and signed by the appropriate study personnel
- Copies of IRB approvals, continuations, and other notifications if appropriate
- Procedure for accounting for missing, unused, and spurious data

# ETHICAL CONSIDERATIONS

TDF is a FDA-approved drug for use in combination with other antiretrovirals for the treatment of HIV infection. Most clinical data suggest a low risk of side effects. However, since this trial involves healthy HIV-uninfected persons, safety will be closely monitored to assess both clinical and behavioral effects. Potential medical risks associated with TDF use include typical side effects associated with all medications, e.g., nausea, rash, etc., or other more serious effects such as creatinine elevations or hypophosphatemia. It is anticipated that prompt discontinuation of study product should quickly result in the resolution of most side effects and that no long-term effects should be experienced by any participant. Long-term safety effects on bone and kidney will be assessed in this study through the use of DEXA scans, laboratory testing, and adverse event reporting.

Another potential clinical and epidemiologic concern is that of antiretroviral resistance. Although, most clinical data suggest the development of the signature K65R mutation occurs infrequently among HIV-infected individuals, it is unclear how often this mutation may occur if TDF is used as monotherapy during early infection when viral levels are elevated and what its subsequent significance for future therapeutic options may be. It is anticipated that the total number of HIV infections in this study will be few, and the consequent number of infections in any arm even fewer. However, in order to detect any HIV infections occurring in this study, HIV testing will be performed at all study visits and on participant request. In addition, an algorithm will be used to detect early infections. Resistance testing and viral set points (CD4 and viral load) will be performed for any persons who become HIV-infected during this study at the time of diagnosis and at repeated intervals to assess the development of resistance and therapeutic response, so as to describe any differences between infection and clinical progression between TDF and placebo recipients.

However, medical risks are not the sole ethical issues of relevance. Participants may have a false perception of being assigned to the TDF arms and of being protected by the study product, possibly leading to increased sexual risk taking. They may also be at risk of personal or social consequences such as embarrassment from answering questions about their sexual behavior or disruption in their personal relationships. These risks will be minimized through community consultation; the development of appropriate community and participant education materials; a strong informed consent process; consistent HIV risk-reduction counseling at each visit; and use of ACASI for sensitive questions (59).

Participants will be compensated for their time, inconvenience, discomfort, and any potential out-of-pocket expenses according to a reimbursement schedule approved by the site’s respective institutions. Due to the long length of follow-up in the study, and the increased cumulative burden associated with many visits over a two-year period, per-visit reimbursement will increase during the second year of the study.

## Community Consultation Process

The intent of the community consultation process is to identify key stakeholders; address major areas of community concern; and establish effective mechanisms for dialogue among community members and researchers (60). This dialogue will emphasize context specific research challenges and strategies to effectively communicate key messages about study design, participant rights, and avoidance of risk taking behaviors. In addition, community consultation will assist researchers in developing recruitment and retention strategies.

Each site has a standing community advisory board (CAB). CABs will be thoroughly briefed about the research. CAB members will provide ongoing advice and commentary about conducting research in their communities, as well as about research procedures and educational materials.

In order to engage the larger advocacy and research community, a variety of consultations may occur at various points during the study. The first consultation occurred in January 2004 and included investigators from ARCA, SFDPH, CDC, and Gilead Sciences, Inc., and representatives from local site CABs and community-based organizations (CBOs). Study investigators led discussions on a host of topics, including study recruitment, changes in risk behavior, occurrence of adverse events, HIV resistance, and study design. Community members commented on study procedures, community context, and ethical issues that were taken into account in the development of this protocol and related materials. Ongoing input is being requested from community members on implementation activities.

## Community and Participant Education

Each site has proven strategies for community education including CABS, community education teams, and use of various media. This trial will capitalize on these existing mechanisms while assuring necessary standardization of messages across sites. Educational materials will be developed that describe key study concepts and participant rights and responsibilities. Educational materials will explain study concepts using accessible and contextually appropriate language and graphics. Key concepts will be reinforced during risk reduction counseling. The ongoing use of multiple written, graphical, and discussion based education techniques has been demonstrated to substantially increase potential participant understanding of complex HIV biomedical interventional trials. (61)

## Informed Consent and Comprehension Test

After presentation of study information at screening, potential participants will be administered a short comprehension test covering key elements of study design and participant rights and responsibilities. These include concepts such as randomization, blinding, withdrawal from study participation, and study medication dosing schedule (Appendix 6). Participants will be required to correctly answer all questions before they can be considered eligible for the trial. The participant will be given two opportunities to pass the test. If he is not successful on the second attempt, he will be ineligible for study participation.

## Risk Reduction Counseling

Standardized risk reduction counseling adapted for the research context, will be used. This multisession client-centered counseling approach emphasizes realistic assessment of one’s own risk and development of gradual, sustainable plan for risk reduction. In order to assure quality and standardization of counseling messages, counselors at both sites will be trained using the same curriculum. (62). Because of community concerns, audio taping or direct observation of counseling sessions for quality assurance is not possible.  However, participants will be asked to complete anonymous surveys about the content of each of their counseling sessions, (Appendix 9). Information from these surveys will be used only at the individual sites. Site counselors will meet regularly with counseling supervisors to discuss issues that have arisen in their counseling sessions and feedback from participant surveys.

# ADMINISTRATIVE STRUCTURE

## Data Management

The trial will be conducted at two clinical sites, using a centralized administrative structure and a centralized data management and analysis process.

## Case Report Forms (CRFs)

CRFs will be supplied by CDC and/or the CRO in paper form or electronically (ACASI, MEMS cap) and should be handled in accordance with instructions from CDC and the CRO. CRF’s requiring participant information are included in Appendix 10.

### Paper data

All paper CRFs will be completed by study staff and submitted to the CRO using the designated procedures.

### Electronic data

All ACASI CRFs will be completed by study participants and stored in a secure database. These records will be transmitted electronically to a central database and verified for accurate interpretation of data. Data from MEMS caps will be downloaded at transmitted electronically according to the manufacturer’s instructions.

## Specimen Collection

Routine laboratory tests, such as electrolytes, blood chemistries, HIV and STI testing will be performed using the sites’ local CLIA approved laboratory facilities. These laboratory data will be maintained in participants’ charts as well as be submitted either electronically or in paper form to the CRO. Blood specimens collected for storage for testing at a later date or for any specialized tests will be sent to a CDC designated central facility.

## Study Initiation

Before the start of this study, the following documents must be submitted to CDC

- Written documentation of site IRB approval of protocol (identified by CDC protocol number or title and date of approval) and informed consent document (identified by CDC protocol number or title and date of approval)
- A copy of the site IRB-approved informed consent documents
- Documentation of IRB review and approval of any advertising materials to be used for study recruitment, if applicable
- Current laboratory certification of the laboratory performing the serum chemistry, electrolyte, and hematologic testing, as well as the current normal laboratory ranges for all laboratory tests

# REFERENCES

1. Joint United Nations Programme on HIV/AIDS WHO. AIDS Epidemic Update: 2003. Available at: <http://www/unaids.org>. (Accessed May 21, 2004).

2. Centers for Disease Control and Prevention. HIV/AIDS Surveillance Report, 2002. Vol 14. 11-3-0003. Atlanta, GA.

3. Jackson JB, Barnett S, Piwowar-Manning E, et al. A phase I/II study of nevirapine for pre-exposure prophylaxis of HIV-1 transmission in uninfected subjects at high risk. AIDS 2003; 17(4):547-553.

4. Guay LA, Musoke P, Fleming T, et al. Intrapartum and neonatal single-dose nevirapine compared with zidovudine for prevention of mother-to-child transmission of HIV-1 in Kampala, Uganda: HIVNET 012 randomised trial. Lancet 1999; 354(9181):795-802.

5. Do AN, Ciesielski CA, Metler RP, Hammett TA, Li J, Fleming PL. Occupationally acquired human immunodeficiency virus (HIV) infection: national case surveillance data during 20 years of the HIV epidemic in the United States. Infect Control Hosp Epidemiol 2003; 24(2):86-96.

6. Cardo DM, Culver DH, Ciesielski CA, et al. A case-control study of HIV seroconversion in health care workers after percutaneous exposure. Centers for Disease Control and Prevention Needlestick Surveillance Group. N Engl J Med 1997; 337(21):1485-1490.

7. Tsai CC, Follis KE, Sabo A, et al. Prevention of SIV infection in macaques by (R)-9-(2-phosphonylmethoxypropyl)adenine. Science 1995; 270(5239):1197-1199.

8. Van Rompay KK, Berardi CJ, Aguirre NL, et al. Two doses of PMPA protect newborn macaques against oral simian immunodeficiency virus infection. AIDS 1998; 12(9):F79-F83.

9. Van Rompay KK, McChesney MB, Aguirre NL, Schmidt KA, Bischofberger N, Marthas ML. Two low doses of tenofovir protect newborn macaques against oral simian immunodeficiency virus infection. J Infect Dis 2001; 184(4):429-438.

10. Otten RA, Smith DK, Adams DR, et al. Efficacy of postexposure prophylaxis after intravaginal exposure of pig-tailed macaques to a human-derived retrovirus (human immunodeficiency virus type 2). J Virol 2000; 74(20):9771-9775.

11. De Maat MM, Ter Heine R, Van Gorp EC, Mulder JW, Mairuhu AT, Beijnen JH. Case series of acute hepatitis in a non-selected group of HIV-infected patients on nevirapine-containing antiretroviral treatment. AIDS 2003; 17(15):2209-2214.

12. Johnson S, Chan J, Bennett CL. Hepatotoxicity after prophylaxis with a nevirapine-containing antiretroviral regimen. Ann Intern Med 2002; 137(2):146-147.

13. Wit FW, Weverling GJ, Weel J, Jurriaans S, Lange JM. Incidence of and risk factors for severe hepatotoxicity associated with antiretroviral combination therapy. J Infect Dis 2002; 186(1):23-31.

14. Centers for Disease Control and Prevention. Serious adverse events attributed to nevirapine regimens for postexposure prophylaxis after HIV exposures--worldwide, 1997-2000. MMWR Morb Mortal Wkly Rep 2001; 49(51-52):1153-1156.

15. Patel SM, Johnson S, Belknap SM, Chan J, Sha BE, Bennett C. Serious adverse cutaneous and hepatic toxicities associated with nevirapine use by non-HIV-infected individuals. J Acquir Immune Defic Syndr 2004; 35(2):120-125.

16. Robbins BL, Srinivas RV, Kim C, Bischofberger N, Fridland A. Anti-human immunodeficiency virus activity and cellular metabolism of a potential prodrug of the acyclic nucleoside phosphonate 9-R-(2-phosphonomethoxypropyl)adenine (PMPA), Bis(isopropyloxymethylcarbonyl)PMPA. Antimicrob Agents Chemother 1998; 42(3):612-617.

17. Balzarini J, Holy A, Jindrich J, et al. Differential antiherpesvirus and antiretrovirus effects of the (S) and (R) enantiomers of acyclic nucleoside phosphonates: potent and selective in vitro and in vivo antiretrovirus activities of (R)-9-(2-phosphonomethoxypropyl)-2,6-diaminopurine. Antimicrob Agents Chemother 1993; 37(2):332-338.

18. Naesens L, Bischofberger N, Augustijns P, et al. Antiretroviral efficacy and pharmacokinetics of oral bis(isopropyloxycarbonyloxymethyl)-9-(2-phosphonylmethoxypropyl)adenine in mice. Antimicrob Agents Chemother 1998; 42(7):1568-1573.

19. Squires K, Pozniak AL, Pierone G, Jr., et al. Tenofovir disoproxil fumarate in nucleoside-resistant HIV-1 infection: a randomized trial. Ann Intern Med 2003; 139(5 Pt 1):313-320.

20. Cheng A, Barriere S, Coakley DF, Chen SS, Wulfsohn M, Toole JJ. Safety profile of Tenofovir DF in antiretroviral experienced patients from randomized, double-blind, placebo-controlled clinical trials. 9^th^ Conference on Retroviruses and Opportunistic Infections; Seattle, WA; Feb. 24-28, 2002.(Abstract 416-W).

21. Gallant JE, Deresinski S. Tenofovir disoproxil fumarate. Clin Infect Dis 2003; 37(7):944-950.

22. Wainberg MA, Miller MD, Quan Y, et al. In vitro selection and characterization of HIV-1 with reduced susceptibility to PMPA. Antivir Ther 1999; 4(2):87-94.

23. Miller MD, Margot N, Lu B, et al. Genotypic and phenotypic predictors of the magnitude of response to tenofovir disoproxil fumarate treatment in antiretroviral-experienced patients. J Infect Dis 2004; 189(5):837-846.

24. White KL, Margot NA, Wrin T, Petropoulos CJ, Miller MD, Naeger LK. Molecular mechanisms of resistance to human immunodeficiency virus type 1 with reverse transcriptase mutations K65R and K65R+M184V and their effects on enzyme function and viral replication capacity. Antimicrob Agents Chemother 2002; 46(11):3437-3446.

25. Miller MD. K65R, TAMs and tenofovir. AIDS Rev 2004; 6(1):22-33.

26. Margot NA, Isaacson E, McGowan I, Cheng A, Miller MD. Extended treatment with tenofovir disoproxil fumarate in treatment-experienced HIV-1-infected patients: genotypic, phenotypic, and rebound analyses. J Acquir Immune Defic Syndr 2003; 33(1):15-21.

27. Farthing C, Khanlou H, Yeh V. Early virologic failure in a pilot study evaluating the efficacy of abacavir, lamivudine and tenofovir in the treatment naive HIV-infected patients. 2nd International AIDS Society Conference on HIV pathogenesis and treatment; Paris, France; Jul 2003. (Abstract 43)

28. Gallant JE, Rodriguez AE, Weinberg, W, et al. Early non-response to tenofovir DF (TDF) + abacavir (ABC) and lamivudine (3TC) in a randomized trial compared to efavirenz (EFV) + ABC and 3TC: ESS30009 unplanned interim analysis. 43rd Interscience Conference on Antimicrobial Agents and Chemotherapy; Chicago, IL; Sept. 14-17, 2003. (Abstract H1722-a).

29. Shere-Wolfe KD, Verley JR. Marked elevation of the creatine phosphokinase level in a patient receiving tenofovir. Clin Infect Dis 2002; 35(9):1137.

30. Murphy MD, O'Hearn M, Chou S. Fatal lactic acidosis and acute renal failure after addition of tenofovir to an antiretroviral regimen containing didanosine. Clin Infect Dis 2003; 36(8):1082-1085.

31. Verhelst D, Monge M, Meynard JL, et al. Fanconi syndrome and renal failure induced by tenofovir: a first case report. Am J Kidney Dis 2002; 40(6):1331-1333.

32. Schaaf B, Aries SP, Kramme E, Steinhoff J, Dalhoff K. Acute renal failure associated with tenofovir treatment in a patient with acquired immunodeficiency syndrome. Clin Infect Dis 2003; 37(3):e41-e43.

33. Creput C, Gonzalez-Canali G, Hill G, Piketty C, Kazatchkine M, Nochy D. Renal lesions in HIV-1-positive patient treated with tenofovir. AIDS 2003; 17(6):935-937.

34. Karras A, Lafaurie M, Furco A, et al. Tenofovir-related nephrotoxicity in human immunodeficiency virus-infected patients: three cases of renal failure, Fanconi syndrome, and nephrogenic diabetes insipidus. Clin Infect Dis 2003; 36(8):1070-1073.

35. Lee JC, Marosok RD. Acute tubular necrosis in a patient receiving tenofovir. AIDS 2003; 17(17):2543-2544.

36. Rollot F, Nazal EM, Chauvelot-Moachon L, et al. Tenofovir-related Fanconi syndrome with nephrogenic diabetes insipidus in a patient with acquired immunodeficiency syndrome: the role of lopinavir-ritonavir-didanosine. Clin Infect Dis 2003; 37(12):e174-e176.

37. Peyriere H, Reynes J, Rouanet I, et al. Renal tubular dysfunction associated with tenofovir therapy: report of 7 cases. J Acquir Immune Defic Syndr 2004; 35(3):269-273.

38. Cihlar T, Birkus G, Greenwalt DE, Hitchcock MJ. Tenofovir exhibits low cytotoxicity in various human cell types: comparison with other nucleoside reverse transcriptase inhibitors. Antiviral Res 2002; 54(1):37-45.

39. Birkus G, Hitchcock MJ, Cihlar T. Assessment of mitochondrial toxicity in human cells treated with tenofovir: comparison with other nucleoside reverse transcriptase inhibitors. Antimicrob Agents Chemother 2002; 46(3):716-723.

40. Rivas P, Polo J, de Gorgolas M, Fernandez-Guerrero ML. Drug points: Fatal lactic acidosis associated with tenofovir. BMJ 2003; 327(7417):711.

41. Lafeuillade A, Jolly P, Chadapaud S, Hittinger G, Lambry V, Philip G. Evolution of lipid abnormalities in patients switched from Stavudine- to tenofovir-containing regimens. J Acquir Immune Defic Syndr 2003; 33(4):544-546.

42. Van Rompay KK, Marthas ML, Lifson JD, et al. Administration of 9-[2-(phosphonomethoxy)propyl]adenine (PMPA) for prevention of perinatal simian immunodeficiency virus infection in rhesus macaques. AIDS Res Hum Retroviruses 1998; 14(9):761-773.

43. Tsai CC, Emau P, Follis KE, et al. Effectiveness of postinoculation (R)-9-(2-phosphonylmethoxypropyl) adenine treatment for prevention of persistent simian immunodeficiency virus SIVmne infection depends critically on timing of initiation and duration of treatment. J Virol 1998; 72(5):4265-4273.

44. Lurie P, Miller S, Hecht F, Chesney MA, Lo B. Postexposure prophylaxis after nonoccupational HIV exposure. JAMA 1998; 280:1769-1773.

45. Blower SM, McLean AR. Prophylactic vaccines, risk behavior change, and the probability of eradicating HIV in San Francisco. Science 1994; 265(5177):1451-1454.

46. Martin J, Roland M, Neilands T, et al. Use of postexposure prophylaxis against HIV infection following sexual exposure does not lead to increases in high-risk behavior. AIDS 2004; 18(5):787-792.

47. Quan VM, Steketee RW, Valleroy L, Weinstock H, Karon J, Janssen R. HIV incidence in the United States, 1978-1999. J Acquir Immune Defic Syndr 2002; 31(2):188-201.

48. Chen SY, Gibson S, Weide D, McFarland W. Unprotected anal intercourse between potentially HIV-serodiscordant men who have sex with men, San Francisco. J Acquir Immune Defic Syndr 2003; 33(2):166-170.

49. James JS. Prevention: new approach will test tenofovir for persons at high risk. AIDS Treat News 2002;(385):4.

50. Cohen J. Cambodia: can a drug provide some protection? Science 2003; 301(5640):1660-1661.

51. Ackers M, Greenberg AE, Lin C, et al. High HIV incidence among HIV vaccine efficacy trial participants. 11^th^ Conference on Retroviruses and Opportunistic Infections; San Francisco, CA; Feb. 8-11, 2004. (Abstract 857).

52. MEMS 6 Medication Monitoring System . http://www.aardex.ch/E/products/sproducts.htm. (Accessed April 23, 2004).

53. Centers for Disease Control and Prevention. Notice to readers: protocols for confirmation of reactive rapid HIV tests. MMWR Morb Mortal Wkly Rep 2004; 53(10):221-222.

54. Hecht FM, Busch MP, Rawal B, et al. Use of laboratory tests and clinical symptoms for identification of primary HIV infection. AIDS 2002; 16(8):1119-1129.

55. Derogatis LR. Brief Symptom Inventory. Baltimore: Clinical Psychometric Research, 1975.

56. Koblin BA, Chesney MA, Husnik MJ, et al. High-risk behaviors among men who have sex with men in 6 US cities: baseline data from the EXPLORE study. Amer J Pub Health 2003; 93(6):926-933.

57. McNabb JJ, Nicolau DP, Stoner JA, Ross J. Patterns of adherence to antiretroviral medications: the value of electronic monitoring. AIDS 2003; 17(12):1763-1767.

58. Walsh JC, Mandalia S, Gazzard BC. Responses to 1 month self-report on adherence to antiretroviral therapy are consistent with electronic data and virological treatment outcome. AIDS 2002; 16(2):269-277.

59. Metzger DS, Koblin B, Turner C, et al. Randomized controlled trial of audio computer-assisted self-interviewing: utility and acceptability in longitudinal studies. Amer J Epidemiol 2000; 152(2):99-106.

60. Morin SF, Maiorana A, Koester KA, Sheon NM, Richards TA. Community consultation in HIV prevention research: a study of community advisory boards at 6 research sites. . J Acquir Immune Defic Syndr 2003; 33(4):513-520.

61. Coletti AS, Heagerty P, Sheon AR, et al. Randomized, controlled evaluation of a prototype informed consent process for HIV vaccine efficacy trials. . J Acquir Immune Defic Syndr 2003; 32(2):161-169.

62. Kamb ML, Dillon BA, Fishbein M, Willis KL, Project RESPECT Study Group. Quality assurance of HIV prevention counseling in a multi-center randomized controlled trial. Public Health Reports 1996; 111(Supplement 1):99-107.

# APPENDICES

## Appendix 1 GILEAD INVESTIGATOR’S BROCHURE

## Appendix 2 GILEAD PACKAGE INSERT

## Appendix 3 STUDY SCHEDULES

Table 1: Study Schedule for HIV-1 Uninfected Participants (Immediate arm) (Part A)

Table 2: Study Schedule for HIV-1 Uninfected Participants (Delayed arm) (Part A)

Table 3: Study Schedule for HIV-1 Infected Participants (Part B)

Appendix 4 FLIP CHART

Appendix 5 STUDY INFORMED CONSENT

Appendix 6 COMPREHENSION ASSESSMENT

Appendix 7 TOXICITY SCALES

Table 1: NIAID Toxicity Scale

Table 2: GILEAD Toxicity Scale

Appendix 8 TOXICITY ALGORITHMS

Table 1: Management of Clinical and Laboratory Adverse Events (Except Creatinine)

Table 2: Management of Creatinine Elevations

Appendix 9 COUNSELING ASSESSMENT

Appendix 10 CASE REPORT FORMS (CRF)

Appendix 11 FOCUS GROUP PROTOCOL

1. Persons with documented history of stable benign neutropenia or documented stable anemia are eligible for participation. [↑](#footnote-ref-2)
2. Only for participants with any resistant virus detected at baseline testing with a HIV RNA PCR > 1000 copies/ml or for participants on antiretroviral therapy with HIV RNA PCR > 1000 copies. [↑](#footnote-ref-3)
